# Harmonized global maps of above and belowground biomass carbon density in the year 2010

**DOI:** 10.1038/s41597-020-0444-4

**Published:** 2020-04-06

**Authors:** Seth A. Spawn, Clare C. Sullivan, Tyler J. Lark, Holly K. Gibbs

**Affiliations:** 1 Department of Geography, University of Wisconsin-Madison, Madison, WI USA; 2 Center for Sustainability and the Global Environment (SAGE), Nelson Institute for Environmental Studies, University of Wisconsin-Madison, Madison, WI USA

**Keywords:** Biogeography, Ecosystem services, Carbon cycle, Carbon cycle, Carbon cycle

## Abstract

Remotely sensed biomass carbon density maps are widely used for myriad scientific and policy applications, but all remain limited in scope. They often only represent a single vegetation type and rarely account for carbon stocks in belowground biomass. To date, no global product integrates these disparate estimates into an all-encompassing map at a scale appropriate for many modelling or decision-making applications. We developed an approach for harmonizing vegetation-specific maps of both above and belowground biomass into a single, comprehensive representation of each. We overlaid input maps and allocated their estimates in proportion to the relative spatial extent of each vegetation type using ancillary maps of percent tree cover and landcover, and a rule-based decision schema. The resulting maps consistently and seamlessly report biomass carbon density estimates across a wide range of vegetation types in 2010 with quantified uncertainty. They do so for the globe at an unprecedented 300-meter spatial resolution and can be used to more holistically account for diverse vegetation carbon stocks in global analyses and greenhouse gas inventories.

## Abstract (summary)

**Table:** 

Measurement(s)	biomass carbon density
Technology Type(s)	digital curation
Factor Type(s)	climatic zone • above or below ground • land cover
Sample Characteristic - Environment	organic material
Sample Characteristic - Location	Earth (planet)

Machine-accessible metadata file describing the reported data: 10.6084/m9.figshare.11872383

## Background & Summary

Terrestrial ecosystems store vast quantities of carbon (C) in aboveground and belowground biomass^1^. At any point in time, these stocks represent a dynamic balance between the C gains of growth and C losses from death, decay and combustion. Maps of biomass are routinely used for benchmarking biophysical models^2–4^, estimating C cycle effects of disturbance^5–7^, and assessing biogeographical patterns and ecosystem services^8–11^. They are also critical for assessing climate change drivers, impacts, and solutions, and factor prominently in policies like Reducing Emissions from Deforestation and Forest Degradation (REDD+) and C offset schemes^12–14^. Numerous methods have been used to map biomass C stocks but their derivatives often remain limited in either scope or extent^12,15^. There thus remains a critical need for a globally harmonized, integrative map that comprehensively reports biomass C across a wide range of vegetation types.

Most existing maps of aboveground biomass (AGB) and the carbon it contains (AGBC) are produced from statistical or data-driven methods relating field-measured or field-estimated biomass densities and spaceborne optical and/or radar imagery^12,15,16^. They largely focus on the AGB of trees, particularly those in tropical landscapes where forests store the majority of the region’s biotic C in aboveground plant matter. Land cover maps are often used to isolate forests from other landcover types where the predictive model may not be appropriate such that forest AGB maps intentionally omit AGB stocks in non-forest vegetation like shrublands, grasslands, and croplands, as well as the AGB of trees located within the mapped extent of these excluded landcovers^17^. Non-forest AGB has also been mapped to some extent using similar approaches but these maps are also routinely masked to the geographic extent of their focal landcover^18–21^. To date, there has been no rigorous attempt to harmonize and integrate these landcover-specific, remotely sensed products into a single comprehensive and temporally consistent map of C in all living biomass.

Maps of belowground biomass (BGB) and carbon density (BGBC) are far less common than those of AGB because BGB cannot be readily observed from space or airborne sensors. Consequently, BGB is often inferred from taxa-, region-, and/or climate-specific “root-to-shoot” ratios that relate the quantity of BGB to that of AGB^22–24^. These ratios can be used to map BGB by spatially applying them to AGB estimates using maps of their respective strata^5^. In recent years, more sophisticated regression-based methods have been developed to predict root-to-shoot ratios of some landcover types based on covariance with other biophysical and/or ecological factors^25,26^. When applied spatially, these methods can allow for more continuous estimates of local BGB^5,27^. Like AGBC, though, few attempts have been made to comprehensively map BGBC for the globe.

Despite the myriad of emerging mapping methods and products, to date, the Intergovernmental Panel on Climate Change (IPCC) Tier-1 maps by Ruesch and Gibbs^28^ remains the primary source of global AGBC and BGBC estimates that transcend individual landcover types. These maps, which represents the year 2000, were produced prior to the relatively recent explosion of satellite-based AGB maps and they therefore rely on an alternative mapping technique called “stratify and multiply”^15^, which assigns landcover-specific biomass estimates or “defaults” (often derived from field measurements or literature reviews) to the corresponding classified grid cells of a chosen landcover map^12^. While this approach yields a comprehensive wall-to-wall product, it can fail to capture finer-scale spatial patterns often evident in the field and in many satellite-based products^12,15^. The accuracy of these maps is also tightly coupled to the quality and availability of field measurements^29^ and the thematic accuracy and discontinuity of the chosen landcover map.

Given the wealth of landcover-specific satellite based AGB maps, a new harmonization method akin to “stratify and multiply” is needed to merge the validated spatial detail of landcover-specific remotely sensed biomass maps into a single, globally harmonized product. We developed such an approach by which we (i) overlay distinct satellite-based biomass maps and (ii) proportionately allocate their estimates to each grid cell (“overlay and allocate”). Specifically, we overlay continental-to-global scale remotely sensed maps of landcover-specific biomass C density and then allocate fractional contributions of each to a given grid cell using additional maps of percent tree cover, thematic landcover and a rule-based decision tree. We implement the new approach here using temporally consistent maps of AGBC as well as matching derived maps of BGBC to generate separate harmonized maps of AGBC and BGBC densities. In addition, we generate associated uncertainty layers by propagating the prediction error of each input dataset. The resulting global maps consistently represent biomass C and associated uncertainty across a broad range of vegetation in the year 2010 at an unprecedented 300 meter (m) spatial resolution.

## Methods

**Fig. 1:**
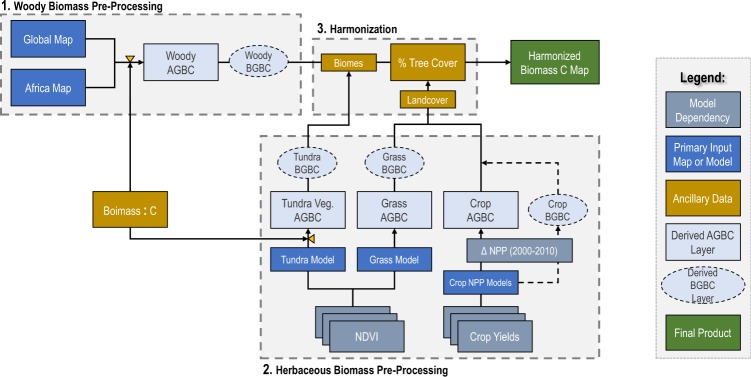
Generalized, three-step workflow used to create harmonized global biomass maps. In step one, woody AGB maps are prepared, combined, converted to AGBC density and used to create separate but complementary maps of BGBC. In step two, a similar workflow is used to generate matching maps of AGBC and BGBC for tundra vegetation, grasses, and annual crops. In step three, all maps are combined using a rule-based decision tree detailed in Fig. 3 to generate comprehensive, harmonized global maps. All input data sources are listed and described in Table 1.

Our harmonization approach (Fig. 1) relies on independent, landcover-specific biomass maps and ancillary layers, which we compiled from the published literature (Table 1). When published maps did not represent our epoch of interest (i.e. grasslands and croplands) or did not completely cover the necessary spatial extent (i.e. tundra vegetation), we used the predictive model reported with the respective map to generate an updated version that met our spatial and temporal requirements. We then used landcover specific root-to-shoot relationships to generate matching BGBC maps for each of the input AGBC maps before implementing the harmonization procedure. Below we describe, in detail, the methodologies used for mapping AGBC and BGBC of each landcover type and the procedure used to integrate them.

**Table 1:** Data sources used to generate harmonized global maps of above and belowground biomass carbon density.

Data source	Description	Use
Santoro *et al*.^30^ (GlobBiomass)	Global, remotely sensed map of woody AGB in living trees with DBH greater than 10 cm and masked to pixels containing Landsat-identified tree cover in 2010^34^. Native resolution of 100 m. Includes accompanying standard error of predictions layer.	Woody AGBC mapping
Bouvet *et al*.^35^	Continental, remotely sensed map of woody AGB in living trees of any size in Africa. Unmasked and includes shrublands. Native resolution of 25 m. RMSE of prediction of 17.0 Mg ha^−1^.	Woody AGBC mapping
CCI Landcover 2010^37^	Landcover map produced at 300 m spatial resolution by the European Space Agency’s Climate Change Initiative. Represents the year 2010.	Woody AGBC mapping; Woody BGBC mapping; Map Harmonization
Xia *et al*.^19^	Non-linear regression relating grassland AGBC density to AVHRR NDVI. Previously used for global mapping. RMSE = 0.3 Mg ha^−1^.	Grassland AGBC mapping
MODIS NDVI (16 Day)^50,51^	16-Day global MODIS Aqua and Terra NDVI composite images. Native resolution of 250 m. Accessed in Google Earth Engine^81^.	Grassland AGBC mapping
Fensholt and Proud^52^	Coefficients for calibrating MODIS to AVHRR NDVI values.	Grassland AGBC mapping
Berner *et al*.^18^	Non-linear regression model relating tundra AGBC density to Landsat ETM derived NDVI. Previously used to map Alaskan Tundra.	Tundra AGBC mapping
MODIS Surface Reflectance (Daily)^46,47^	Daily global NDVI composite images derived from MODIS Aqua and Terra surface reflectance images. Native resolution of 250 m. Accessed in Google Earth Engine^81^.	Tundra AGBC mapping
Steven *et al*.^48^	Coefficients for calibrating MODIS to Landsat ETM NDVI values.	Tundra AGBC mapping
Monfreda *et al*.^20^	Globally gridded yield maps for 70 annually harvested herbaceous commodity crops (Online-only Table 2) representing c. 2000 at 8 km resolution. Crop-specific parameters used to calculate cropland ANPP.	Cropland AGBC mapping; Cropland BGBC mapping
Wolf *et al*.^21^	Crop-specific parameters and model used to calculate cropland ANPP.	Cropland AGBC mapping; Cropland BGBC mapping
Ramankutty *et al*.^55^	Map of global cropland area c. 2000 that complements the global crop yield maps of Monfreda *et al*.^20^ at 8 km resolution.	Cropland AGBC mapping; Cropland BGBC mapping
MODIS ANPP^58^	Remotely sensed global maps of modelled MODIS Terra ANPP (2000–2015) at 1 km native resolution. Accessed in Google Earth Engine^81^.	Cropland AGBC mapping; Cropland BGBC mapping
Reich *et al*.^25^	Multiple regression model predicting BGB of trees from AGB using environmental covariates.	Woody BGBC mapping
Potopov *et al*.^60^	Global map of “Intact forested landscapes” which are defined as large contiguous forest patches not influenced by human activity. User’s accuracy = 92%.	Woody BGBC mapping
Harris *et al*.^61^	Spatial database of planted trees with incomplete global coverage	Woody BGBC mapping
FRA^62^	FAO Global Forest Resource Assessment – national statistics on the spatial extent of natural and planted forests and other woody vegetation.	Woody BGBC mapping
FAOSTAT	FAOSTAT database – national statistics on the planted area of tree crops. (http://www.fao.org/faostat)	Woody BGBC mapping
Fick and Hijmans^59^ (WorldClim version 2)	Global map of mean annual temperature (MAT) between 1980–2000. 1 km native resolution. RMSE = 1.12 °C.	Woody BGBC mapping; Tundra BGBC mapping
Wang *et al*.^26^	Regression model predicting the root-to-shoot ratios of tundra plants from MAT.	Tundra BGBC mapping
Mokany *et al*.^22^	Mean and standard error of field-measured root-to-shoot ratios for natural landcover types, stratified by climatic zone.	Woody BGBC mapping Grassland BGBC mapping;
Kottek *et al*.^43^	Updated version of the Köppen-Gieger climate classification.	Woody AGBC mapping; Woody BGBC mapping; Grassland BGBC mapping
Martin *et al*.^42^	Mean and standard error of field measured biomass carbon fractions globally stratified by climatic zone and plant phylogeny.	Woody AGBC mapping; Woody BGBC mapping; Tundra AGBC mapping; Tundra BGBC mapping
MODIS Treecover^63^	Global map of percent tree cover in 2010 from MODIS Terra at a native 250 m resolution. Includes estimated standard deviation of each grid cell’s prediction. Accessed in Google Earth Engine^81^.	Map Harmonization
Resolve2017 Biomes^65^	Updated polygonal extents of the Olson biome classification^83^. Accessed in Google Earth Engine^81^.	Map Harmonization

### Aboveground biomass carbon density maps

#### Woody tree biomass

Since the first remotely sensed woody AGB maps were published in the early 1990s, the number of available products has grown at an extraordinary pace^16^ and it can thus be challenging to determine which product is best suited for a given application. For our purposes, we relied on the GlobBiomass AGB density map^30^ as our primary source of woody AGB estimates due to its precision, timestamp, spatial resolution, and error quantification. It was produced using a combination of spaceborne optical and synthetic aperture radar (SAR) imagery and represents the year 2010 at a 100 m spatial resolution – making it the most contemporary global woody AGB currently available and the only such map available for that year. Moreover, GlobBiomass aims to minimize prediction uncertainty to less than 30% and a recent study suggests that it has high fidelity for fine-scale applications^31^.

The GlobBiomass product was produced by first mapping the growing stock volume (GSV; i.e. stem volume) of living trees, defined following Food and Agriculture Organization (FAO) guidelines^32^ as those having a diameter at breast height (DBH) greater than 10 centimeters (cm). AGB density was then determined from GSV by applying spatialized biomass expansion factors (BEFs) and wood density estimates. These factors were mapped using machine learning methods trained from a suite of plant morphological databases that compile thousands of field measurements from around the globe^33^. The resulting AGB estimates represent biomass in the living structures (stems, branches, bark, twigs) of trees with a DBH greater than 10 cm. This definition may thereby overlook AGB of smaller trees and/or shrubs common to many global regions. Unlike other maps, though, the GlobBiomass product employs a subpixel masking procedure that retains AGB estimates in 100 m grid cells in which any amount of tree cover was detected in finer resolution (30 m) imagery^34^. This unique procedure retains AGB estimates in tree-sparse regions like savannahs, grasslands, croplands, and agroforestry systems where AGB is often overlooked^17^, as well as in forest plantations. The GlobBiomass product is the only global map that also includes a dedicated uncertainty layer reporting the standard error of prediction. We used this layer to propagate uncertainty when converting AGB to AGBC density, modelling BGBC, and integrating with C density estimates of other vegetation types.

Bouvet *et al*.^35^ – some of whom were also participants of the GlobBiomass project – independently produced a separate AGB density map for African savannahs, shrublands and dry woodlands circa 2010 at 25 m spatial resolution^35^ (hereafter “Bouvet map”), which we included in our harmonized product to begin to address the GlobBiomass map’s potential omission of small trees and shrubs that do not meet the FAO definition of woody AGB. This continental map of Africa is based on a predictive model that directly relates spaceborne L-band SAR imagery – an indirect measure of vegetation structure that is sensitive to low biomass densities^36^ – with region-specific, field-measured AGB. Field measurements (n = 144 sites) were compiled from 7 different sampling campaigns – each specifically seeking training data for biomass remote sensing – that encompassed 8 different countries^35^. The resulting map is not constrained by the FAO tree definition and is masked to exclude grid cells in which predicted AGB exceeds 85 megagrams dry mater per hectare (Mg ha^−1^) – the threshold at which the SAR-biomass relationship saturates. To avoid extraneous prediction, it further excludes areas identified as “broadleaved evergreen closed-to-open forest”, “flooded forests”, “urban areas” and “water bodies” by the European Space Agency’s Climate Change Initiative (CCI) Landcover 2010 map^37^ and as “bare areas” in the Global Land Cover (GLC) 2000 map^38^. While the Bouvet map is not natively accompanied by an uncertainty layer, its authors provided us with an analytic expression of its uncertainty (*SE*; standard error of prediction) as a function of estimated AGB (Eq. 1) which we used to generate an uncertainty layer for subsequent error propagation.

1 $$SE=1.0551\cdot AGB-0.007\cdot AG{B}^{2}-0.0000273\cdot AG{B}^{3};\,0\le AGB\le 85\,{\rm{Mg}}\,{{\rm{ha}}}^{-1}$$

**Fig. 2:**
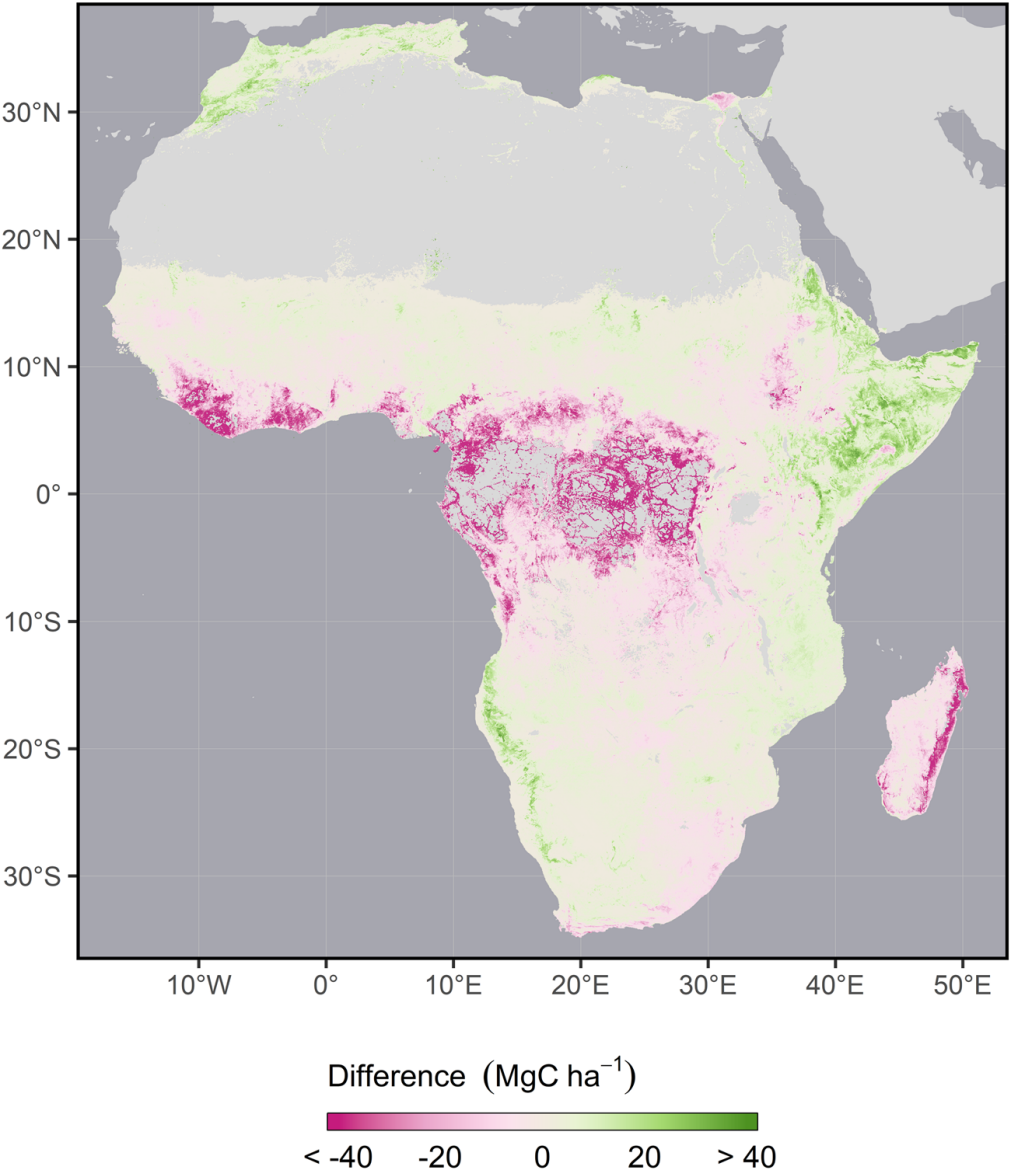
Difference between underlying woody aboveground biomass maps in Africa. Maps considered are the GlobBiomass^30^ global map and the Bouvet^35^ map of Africa. Both maps were aggregated to a 300 m spatial resolution and converted to C density prior to comparison using the same schema. The difference map was subsequently aggregated to a 3 km spatial resolution and reprojected for visualization. Negative values denote lower estimates by Bouvet *et al*.^35^, while positive values denote higher estimates.

We combined the GlobBiomass and Bouvet products to generate a single woody biomass map by first upscaling each map separately to a matching 300 m spatial resolution using an area-weighted average to aggregate grid cells, and then assigning the Bouvet estimate to all overlapping grid cells, except those identified by the CCI Landcover 2010 map as closed or flooded forest types (Online-only Table 1) which were not within the dryland domain of the Bouvet map. While more complex harmonization procedures based on various averaging techniques have been used by others^39,40^, their fidelity remains unclear since they fail to explicitly identify and reconcile the underlying source of the inputs’ discrepancies^41^. We thus opted to use a more transparent ruled-based approach when combining these two woody biomass maps, which allows users to easily identify the source of a grid cell’s woody biomass estimate. Given the local specificity of the training data used to produce the Bouvet map, we chose to prioritize its predictions over those of the GlobBiomass product when within its domain. In areas of overlap, the Bouvet map values tend to be lower in moist regions and higher in dryer regions (Fig. 2), though, where used, these differences rarely exceed ±25 megagrams C per hectare (MgC ha^−1^).

We then converted all woody AGB estimates to AGBC by mapping climate and phylogeny-specific biomass C concentrations from Martin *et al*.^42^. Climate zones were delineated by aggregating classes of the Köppen-Gieger classification^43^ (Table 2) to match those of Martin *et al*.^42^. Phylogenetic classes (angiosperm, gymnosperm and mixed/ambiguous) were subsequently delineated within each of these zones using aggregated classes of the CCI Landcover 2010 map (Online-only Table 1). Martin *et al*.^42^ only report values for angiosperms and gymnosperms so grid cells with a mixed or ambiguous phylogeny were assigned the average of the angiosperm and gymnosperm values and the standard error of this value was calculated from their pooled variance. Due to residual classification error in the aggregated phylogenetic classes, we weighted the phylogeny-specific C concentration within each climate zone by the binary probability of correctly mapping that phylogeny (i.e. user’s accuracy)^44^ using Eq. 2

2 $${\mu }_{c}={\mu }_{m}{p}_{m}+\frac{({\mu }_{n}+{\mu }_{o})}{2}\left(1-{p}_{m}\right)$$

where, within each climate zone, *μ*_*c*_ is the mean probability-weighted C concentration of the most probable phylogeny, *μ*_*m*_ is the mean C concentration of that phylogeny from Martin *et al*.^42^, *p*_*m*_ is the user’s accuracy of that phylogeny’s classification (Table 3), and *μ*_*n*_ and *μ*_*o*_ are the mean C concentrations of the remain phylogenetic classes from Martin *et al*.^42^. Standard error estimates for these C concentrations were similarly weighted using summation in quadrature (Eq. 3)

3 $${\sigma }_{c}=\sqrt{2{\sigma }_{m}^{2}+{\left(\frac{1-{p}_{m}}{2}{\sigma }_{n}\right)}^{2}+{\left(\frac{1-{p}_{m}}{2}{\sigma }_{o}\right)}^{2}+{({p}_{m}{\sigma }_{m})}^{2}}$$

where ${\sigma }_{c}$ is the probability-weighted standard error of the most probable phylogeny’s C concentration and ${\sigma }_{m}$, ${\sigma }_{n}$ and ${\sigma }_{o}$ are the standard errors of the respective phylogeny-specific C concentrations from Martin *et al*.^42^. Probability-weighted C concentrations used are reported in Table 4.

**Table 2:** Reclassification table of the Köppen-Gieger climate classification.

KG Code	KG Class	KG Main Climate	KG Precipitation	KG Temperature	Mokany *et al*.^22^ Grassland Class	Martin *et al*.^42^ Carbon Domain
1	Af	Equatorial	Fully Humid	—	Tropical/Subtropical	Tropical
2	Am	Equatorial	Monsoonal	—	Tropical/Subtropical	Tropical
3	As	Equatorial	Summer Dry	—	Tropical/Subtropical	Tropical
4	Aw	Equatorial	Winter Dry	—	Tropical/Subtropical	Tropical
5	BSh	Arid	Steppe	Hot Arid	Temperate	Subtropical/Mediterranean
6	BSk	Arid	Steppe	Cold Arid	Temperate	Temperate
7	BWh	Arid	Desert	Hot Arid	Temperate	Subtropical/Mediterranean
8	BWk	Arid	Desert	Cold Arid	Temperate	Temperate
9	Cfa	W. Temperate	Fully Humid	Hot Summer	Temperate	Subtropical/Mediterranean
10	Cfb	W. Temperate	Fully Humid	Warm Summer	Temperate	Temperate
11	Cfc	W. Temperate	Fully Humid	Cool Summer	Temperate	Temperate
12	Csa	W. Temperate	Summer Dry	Hot Summer	Temperate	Subtropical/Mediterranean
13	Csb	W. Temperate	Summer Dry	Warm Summer	Temperate	Temperate
14	Csc	W. Temperate	Summer Dry	Cool Summer	Temperate	Temperate
15	Cwa	W. Temperate	Winter Dry	Hot Summer	Temperate	Subtropical/Mediterranean
16	Cwb	W. Temperate	Winter Dry	Warm Summer	Temperate	Subtropical/Mediterranean
17	Cwc	W. Temperate	Winter Dry	Cool Summer	Temperate	Temperate
18	Dfa	Snow	Fully Humid	Hot Summer	Cool Temperate	Temperate
19	Dfb	Snow	Fully Humid	Warm Summer	Cool Temperate	Temperate
20	Dfc	Snow	Fully Humid	Cool Summer	Tundra	Boreal
21	Dfd	Snow	Fully Humid	Ext. Continental	Tundra	Boreal
22	Dsa	Snow	Summer Dry	Hot Summer	Cool Temperate	Temperate
23	Dsb	Snow	Summer Dry	Warm Summer	Cool Temperate	Temperate
24	Dsc	Snow	Summer Dry	Cool Summer	Cool Temperate	Boreal
25	Dsd	Snow	Summer Dry	Ext. Continental	Cool Temperate	Boreal
26	Dwa	Snow	Winter Dry	Hot Summer	Cool Temperate	Temperate
27	Dwb	Snow	Winter Dry	Warm Summer	Cool Temperate	Temperate
28	Dwc	Snow	Winter Dry	Cool Summer	Tundra	Boreal
29	Dwd	Snow	Winter Dry	Ext. Continental	Tundra	Boreal
30	EF	Polar	—	Polar Frost	Tundra	Boreal
31	ET	Polar	—	Polar Tundra	Tundra	Boreal
32	Ocean	—	—	—	Tropical/Subtropical	Global

The Köppen-Gieger (KG) climate classification^43^ was used to stratify grassland root-to-shoot ratios from Mokany *et al*.^22^ as described in Table 5 and biomass carbon concentrations by Martin *et al*.^42^ as described in Table 4.

**Table 3:** Area-weighted confusion matrix for “likely forest phylogeny” classes.

	Gymno.	Mixed	Angio.	User’s Acc.
Gymno.	0.0180	0.0124	0.0024	55%
Mixed	0.0020	0.9241	0.0086	99%
Angio.	0.0006	0.0076	0.0243	75%
Prod. Acc.	87%	98%	69%	**Overall**: 97%

Classes were aggregated from the CCI landcover map^37^ (Online-only Table 1) and the associated confusion matrix for the year 2010 (Tables 4–5 in version 2.5 of the D3.4-PUG CCI Landcover Product User Guide^84^). User’s accuracies were used to propagate uncertainty of phylogenetic classification when converting biomass density to carbon density and in woody BGBC calculations.

**Table 4:** Climate and phylogeny specific biomass C fractions used to convert biomass density estimates to carbon density.

Climatic Zone	Phylogeny	Mean	SE
Tropical	Angio.	0.454	0.003
	Mixed	0.452	0.004
	Gymno	0.450	0.008
Subtropical/Mediterranean	Angio.	0.465	0.006
	Mixed	0.478	0.008
	Gymno	0.484	0.009
Temperate	Angio.	0.472	0.005
	Mixed	0.483	0.006
	Gymno	0.489	0.006
Boreal	Angio.	0.488	0.013
	Mixed	0.480	0.011
	Gymno	0.476	0.009
Global	Angio.	0.471	0.011
	Mixed	0.476	0.016
	Gymno	0.479	0.012

C fractions were taken from Martin *et al*.^42^ and weighted by the aggregated probability of correct phylogenetic classification (i.e. user’s accuracy) from Table 3. Climate zones are spatially defined in Table 2.

Mapped, probability-weighted C estimates were then arithmetically applied to AGB estimates. Uncertainty associated with this correction was propagated using summation in quadrature of the general form (Eq. 4)

4 $${\sigma }_{f}=\sqrt{{\left(\frac{\partial f}{\partial i}{\sigma }_{i}\right)}^{2}+{\left(\frac{\partial f}{\partial j}{\sigma }_{j}\right)}^{2}+\ldots +{\left(\frac{\partial f}{\partial k}{\sigma }_{k}\right)}^{2}}$$

where ${\mu }_{f}=f(i,j,\ldots ,k)$, ${\sigma }_{f}$ is the uncertainty of *μ*_*f*_, and ${\sigma }_{i},{\sigma }_{j},\ldots ,{\sigma }_{k}$, are the respective uncertainty estimates of the dependent parameters (standard error unless otherwise noted). Here, *μ*_*f*_, is the estimated AGBC of a given grid cell, and is the product of its woody AGB estimate, and its corresponding C concentration.

#### Tundra vegetation biomass

The tundra and portions of the boreal biome are characterized by sparse trees and dwarf woody shrubs as well as herbaceous cover that are not included in the GlobBiomass definition of biomass. AGB density of these classes has been collectively mapped by Berner *et al*.^18,45^ for the North Slope of Alaska from annual Landsat imagery composites of the normalized difference vegetation index (NDVI) and a non-linear regression-based model trained from field measurements of peak AGB that were collected from the published literature (n = 28 sites). Berner *et al*.^18^ note that while these field measurements did not constitute a random or systematic sample, they did encompass a broad range of tundra plant communities. In the absence of a global map and due the sparsity of high quality Landsat imagery at high latitudes, we extended this model to the pan-Arctic and circumboreal regions using NDVI composites created from daily 250 m MODIS Aqua and Terra surface reflectance images^46,47^ that were cloud masked and numerically calibrated to Landsat ETM reflectance – upon which the tundra model is based – using globally derived conversion coefficients^48^. We generated six separate 80^th^ percentile NDVI composites circa 2010 – one for each of the MODIS missions (Aqua and Terra) in 2009, 2010 and 2011 – following Berner *et al*.^18^. We chose to use three years of imagery (circa 2010) rather than just one (2010) to account for the potential influence that cloud masking may exert upon estimates of the 80^th^ NDVI percentile in a single year. We then applied the tundra AGB model to each composite, converted AGB estimates to AGBC by assuming a biomass C fraction of 49.2% (SE = 0.8%)^42^ and generated error layers for each composite from the reported errors of the AGB regression coefficients and the biomass C conversion factor using summation in quadrature as generally described above (Eq. 4). A single composite of tundra AGBC circa 2010 was then created as the pixelwise mean of all six composites. We also generated a complementary uncertainty layer representing the cumulative standard error of prediction, calculated as the pixelwise root mean of the squared error images in accordance with summation in quadrature. Both maps were upscaled from their native 250 m spatial resolution to a 300 m spatial resolution using an area weighted aggregation procedure, whereby pixels of the 300 m biomass layer was calculated as the area weighted average of contained 250 m grid cells, and the uncertainty layer was calculated – using summation in quadrature – as the root area-weighted average of the contained grid cells squared.

#### Grassland biomass

Grassland AGBC density was modelled directly from maximum annual NDVI composites using a non-linear regression-based model developed by Xia *et al*.^19^ for mapping at the global scale. This model was trained by relating maximum annual NDVI as measured by the spaceborne Advanced Very High-Resolution Radiometer (AVHRR) sensor to globally distributed field measurements of grassland AGBC that were compiled from the published literature (81 sites for a total of 158 site-years). Like the tundra biomass training data, these samples did not constitute a random or systematic sample but do encompass a comprehensive range of global grassland communities. Given the inevitable co-occurrence of trees in the AVHRR sensor’s 8 km resolution pixels upon which the model is trained, it’s predictions of grassland AGBC are relatively insensitive to the effects of co-occurring tree cover. We thereby assume that its predictions for grid cells containing partial tree cover represent the expected herbaceous AGBC density in the absence of those trees. Maximum model predicted AGBC (NDVI = 1) is 2.3 MgC ha^−1^ which is comparable to the upper quartile of herbaceous AGBC estimates from global grasslands^49^ and suggests that our assumption will not lead to an exaggerated estimation. For partially wooded grid cells, we used modelled grassland AGBC density to represent that associated with the herbaceous fraction of the grid cell in a manner similar to Zomer *et al*.^17^ as described below (See “*Harmonizing Biomass Carbon Maps”)*.

We applied the grassland AGBC model to all grid cells of maximum annual NDVI composites produced from finer resolution 16-day (250 m) MODIS NDVI imagery composites circa 2010^50,51^. Here again, three years of imagery were used to account for potential idiosyncrasies in a single year’s NDVI composites resulting from annual data availability and quality. As with AGB of tundra vegetation, annual composites (2009–2011) were constructed separately from cloud-masked imagery collected by both MODIS missions (Aqua and Terra; n = 6) and then numerically calibrated to AVHRR reflectance using globally derived conversion coefficients specific to areas of herbaceous cover^52^. We then applied the AGBC model to each of these composites and estimated error for each composite from both the AVHRR calibration (standard deviation approximated from the 95% confidence interval of the calibration scalar) and the AGBC model (relative RMSE) using summation in quadrature. A single map of grassland AGBC circa 2010 was then created as the pixelwise mean of all six composites and an associated error layer was created as the pixelwise root mean of the squared error images. Both maps were aggregated from their original 250 m resolution to 300 m to facilitate harmonization using the area-weighted procedure described previously for woody and tundra vegetation (see section 1.2).

#### Cropland biomass

Prior to harvest, cropland biomass can also represent a sizable terrestrial C stock. In annually harvested cropping systems, the maximum standing biomass of these crops can be inferred from annual net primary productivity (ANPP). While spaceborne ANPP products exist, they generally perform poorly in croplands^53,54^. Instead, cropland ANPP is more commonly derived from crop yields^20,21,53^. We used globally gridded, crop-specific yields of 70 annually harvested herbaceous commodity crops circa 2000 by Monfreda *et al*.^20^ – the only year in which these data were available. These maps were produced by spatially disaggregating crop-yield statistics for thousands of globally distributed administrative units throughout the full extent of a satellite-based cropland map^20^. These maps were combined with crop-specific parameters (Online-only Table 2) to globally map AGBC as aboveground ANPP for each crop following the method of Wolf *et al*.^21^. This method can be simplified as (Eq. 5)

5 $$AGBC=y\omega (0.451{h}^{-1}+1.025c-0.451)$$

where *y* is the crop’s yield (Mg ha^−1^), *ω* is the dry matter fraction of its harvested biomass, *h* is its harvest index (fraction of total AGB collected at harvest) and *c* is the carbon content fraction of its harvested dry mass. This simplification assumes, following Wolf *et al*.^21^, that 2.5% of all harvested biomass is lost between the field and farmgate and that unharvested residue and root mass is 44% C.

Total cropland AGBC density was then calculated as the harvested-area-weighted average of all crop-specific AGBC estimates within a given grid cell. Since multiple harvests in a single year can confound inference of maximum AGBC from ANPP, we further determined the harvest frequency (*f*) of each grid cell by dividing a cell’s total harvested area (sum of the harvested area of each crop reported within a given grid cell) by its absolute cropland extent as reported in a complementary map by Ramankutty *et al*.^55^. If *f* was greater than one, multiple harvests were assumed to have occurred and AGBC was divided by *f* to ensure that AGBC estimates did not exceed the maximum standing biomass density.

Since the yields of many crops and, by association, their biomass have changed considerably since 2000^56,57^, we calibrated our circa 2000 AGBC estimates to the year 2010 using local rates of annual ANPP change (MgC ha^−1^ yr^−1^) derived as the Theil-Sen slope estimator – a non-parametric estimator that is relatively insensitive to outliers – of the full MODIS Terra ANPP timeseries (2000–2015)^58^. Total ANPP change between 2000 and 2010 for each grid cell was calculated as ten times this annual rate of change. Since MODIS ANPP represents C gains in both AGB and BGB, we proportionately allocated aboveground ANPP to AGBC using the total root-to-shoot ratio derived from the circa 2000 total crop AGBC and BGBC maps (described below). Since error estimates were not available for the yield maps or the crop-specific parameters used to generate the circa 2000 AGBC map, estimated error of the circa 2010 crop AGBC map was exclusively based on that of the 2000–2010 correction. The error of this correction was calculated as the pixel-wise standard deviation of bootstrapped simulations (n = 1000) in which a random subset of years was omitted from the slope estimator in each iteration. The 8 km resolution circa 2000 AGBC map and error layer were resampled to 1 km to match the resolution of MODIS ANPP using the bilinear method prior to ANPP correction and then further resampled to 300 m to facilitate harmonization.

Woody crops like fruit, nut, and palm oil plantations were not captured using the procedure just described and their biomass was instead assumed to be captured by the previously described woody biomass products which retained biomass estimates in all pixels where any amount of tree cover was detected at the sub-pixel level (see section 1.1).

### Belowground biomass carbon maps

Matching maps of BGBC and associated uncertainty were subsequently produced for each of the landcover-specific AGBC maps using published empirical relationships.

With the exception of savannah and shrubland areas, woody BGBC was modelled from AGBC using a multiple regression model by Reich *et al*.^25^ that considers the phylogeny, mean annual temperature (MAT), and regenerative origin of each wooded grid cell and that was applied spatially using maps of each covariate in a fashion similar to other studies^5,27^. Tree phylogeny (angiosperm or gymnosperm) was determined from aggregated classes of the CCI Landcover 2010 map^37^ (Online-only Table 1) with phylogenetically mixed or ambiguous classes assumed to be composed of 50% of each. MAT was taken from version 2 of the WorldClim bioclimatic variables dataset (1970–2000) at 1 km resolution^59^ and resampled to 300 m using the bilinear method. Since there is not a single global data product mapping forest management, we determined tree origin – whether naturally propagated or planted – by combining multiple data sources. These data included (i) a global map of “Intact Forest Landscapes” (IFL) in the year 2013^60^ (a conservative proxy of primary, naturally regenerating forests defined as large contiguous areas with minimal human impact), (ii) a Spatial Database of Planted Trees (SDPT) with partial global coverage^61^, (iii) national statistics reported by the FAO Global Forest Resources Assessment (FRA) on the extent of both naturally regenerating and planted forests and woodlands within each country in the year 2010^62^, and (iv) national statistics reported by the FAOSTAT database (http://www.fao.org/faostat) on the planted area of plantation crops in 2010. Within each country, we assumed that the total area of natural and planted trees was equal to the corresponding FRA estimates. If the FAOSTAT-reported area of tree crops exceeded FRA-reported planted area, the difference was added to FRA planted total. All areas mapped as IFL were assumed to be of natural origin and BGB was modelled as such. Likewise, besides the exceptions noted below, all tree plantations mapped by the SDPT were assumed to be of planted origin. In countries where the extent of the IFL or SDPT maps fell short of the FRA/FAOSTAT reported areas of natural or planted forests, respectively, we estimated BGBC in the remaining, unknown-origin forest grid cells of that country (*BGBC*_*u*_), as the probability-weighted average of the planted and natural origin estimates using Eq. 6

6 $$BGB{C}_{u}=BGB{C}_{p}\left(\frac{{\Delta }_{p}}{{\Delta }_{p}+{\Delta }_{n}}\right)+BGB{C}_{n}\left(\frac{{\Delta }_{n}}{{\Delta }_{p}+{\Delta }_{n}}\right)$$

where $BGB{C}_{p}$ and $BGB{C}_{n}$ are the respective BGBC estimates for a grid cell assuming entirely planted and natural origin, respectively, and ${\Delta }_{p}$ and ${\Delta }_{n}$ are the respective differences between (i) the FRA/FAOSTAT and (ii) mapped extent of planted and natural forest within the given grid cell’s country. While the mapped extent of IFL forests within a given country never exceeded that country’s FRA reported natural forest extent, there were infrequent cases (n = 22 of 257) in which the mapped extent of tree plantations exceeded the corresponding FRA/FAOSTAT estimate of planted forest area. In these cases, we down-weighted the BGB estimates of SDPT forests in a similar fashion such that the weight of their planted estimate (${\omega }_{p}$) was equal to the quotient of (i) the FRA/FAOSTAT planted area and (ii) the SDPT extent within the country, and the weight of the natural origin estimate applied to the SDPT extent (${\omega }_{n}$) was equal to $1-{\omega }_{p}$.

A BGBC error layer was then produced using summation in quadrature from the standard error estimates of the model coefficients, the AGBC error layer, the relative RMSE of MAT (27%), and the derived global uncertainty of the phylogeny layer. Phylogeny error was calculated as the Bernoulli standard deviation (*δ*) of the binary probability (*p*) of correct classification (i.e. “area weighted user’s accuracy”^44^; Table 3) using Eq. 7.

7 $$\delta =\sqrt{p\left(1-p\right)}$$

Since savannahs and shrublands are underrepresented in the regression-based model^25^, their BGBC was instead estimated using static root-to-shoot ratios reported by Mokany *et al*.^22^, which are somewhat conservative in comparison to the IPCC Tier-1 defaults^23,24^ put favoured for consistency with methods used for grasslands (see below). Error was subsequently mapped from that of the AGBC estimates and the root-to-shoot ratios applied (Table 5).

**Table 5:** Root-to-shoot ratios used to map BGB of select landcover types.

Taxa	Climate	Strata/Taxa Map	Mean	SE
Savannah	All	CCI Landcover	0.642	0.111
Shrub	All	CCI Landcover	1.837	0.589
Grassland	Tropical/Subtropical	Köppen-Gieger	1.887	0.304
	Temperate	Köppen-Gieger	4.224	0.518
	Cool Temperate	Köppen-Gieger	4.504	1.337
	Tundra	Köppen-Gieger	4.804	1.188

Root-to-shoot ratios and their standard errors were taken from Mokany *et al*.^22^. Grassland stratification classes correspond with those reported as “Mokany Grassland Class” in Table 2.

BGBC of tundra vegetation was mapped from AGBC using a univariate regression model derived by Wang *et al*.^26^ that predicts root-to-shoot ratio as a function of MAT. We applied the model using the WorldClim version 2 MAT map^59^ and propagated error from the AGBC estimates, the relative RMSE of MAT and the standard error of regression coefficients. Where tundra AGB exceeded 25 Mg ha^−1^ – the maximum field-measured shrub biomass reported by Berner *et al*.^18^ – vegetation was considered to include trees and the Reich *et al*.^25^ method described earlier for woody vegetation was used instead.

In the absence of a continuous predictor of grassland root-to-shoot ratios, we applied climate specific root-to-shoot ratios from Mokany *et al*.^22^ to the corresponding climate regions of the Köppen-Gieger classification^43^ (Table 2). Here, again, these ratios vary slightly from the IPCC Tier-1 defaults^23,24^ but were chosen for their greater sample size and specificity. Grassland BGBC error was mapped from the error of the AGBC estimates and the respective root-to-shoot ratios.

Cropland BGBC was again estimated from crop-specific yields and morphological parameters (Online-only Table 2) following Wolf *et al*.^21^ and Eq. 8

8 $$BGBC=0.451\,yr{h}^{-1}$$

where *y* is the crop’s yield (Mg ha^−1^), *r* is the root-to-shoot ratio of the crop, and *h* is its harvest index. Here again we assume that 2.5% of all harvested biomass is lost between the field and farmgate and that root biomass is 44% C, following Wolf *et al*.^21^. BGBC error was mapped from the error of the 2000-to-2010 ANPP correction for BGBC allocation as described above for cropland AGBC.

### Harmonizing biomass carbon maps

**Fig. 3:**
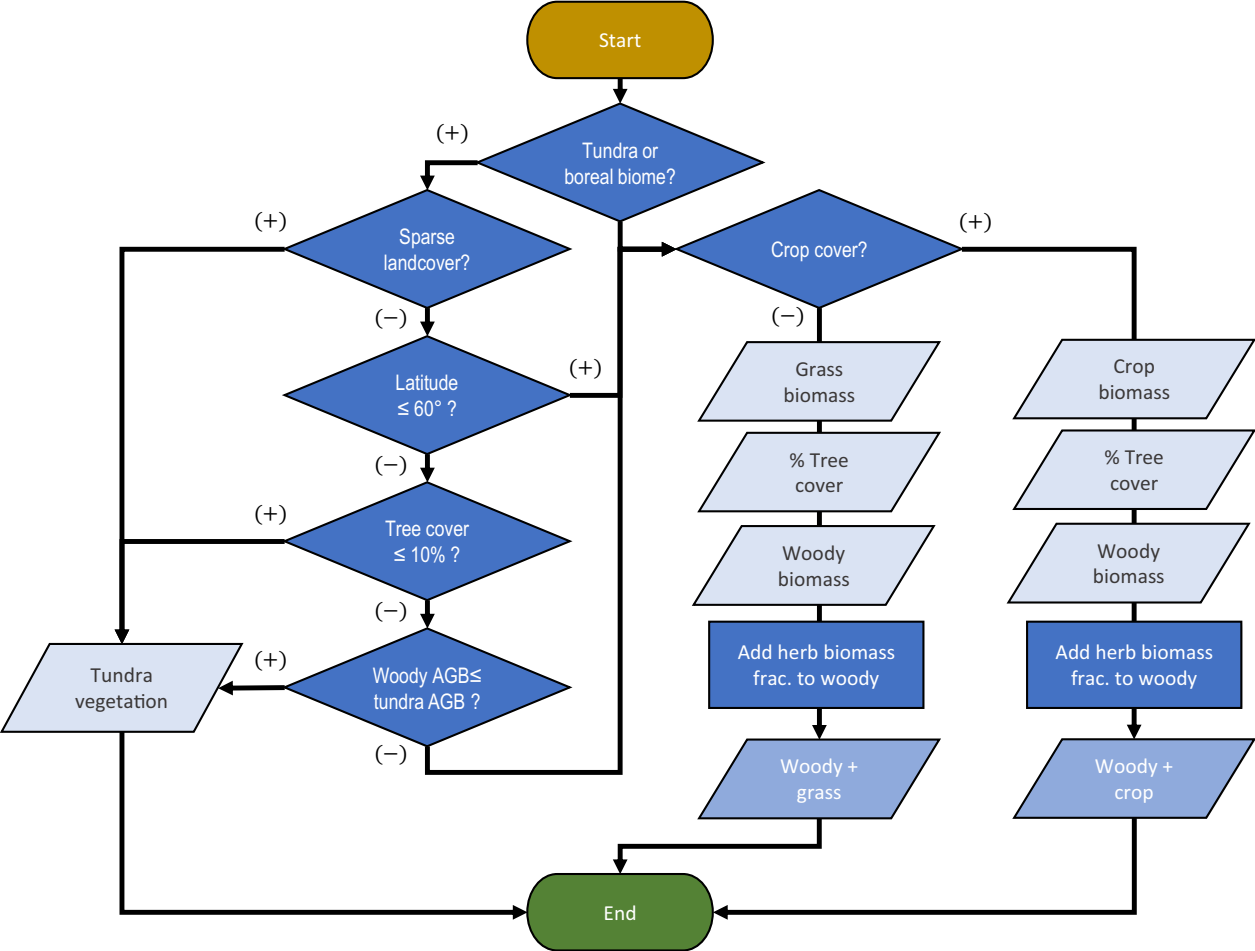
Decision tree used to allocate landcover-specific biomass estimates to each grid cell of our harmonized global products.

The AGBC and BGBC maps were harmonized separately following the same general schema (Fig. 3). Given that our harmonized woody biomass map contains biomass estimates for grid cells in which any amount of tree cover was detected at the subpixel level (see section 1.1), we conserved its estimates regardless of the landcover reported by the 2010 CCI map in order to more fully account for woody biomass in non-forested areas^17^. We then used the MODIS continuous vegetation fields percent tree cover map for 2010^63^ to allocate additional biomass density associated with the most probable herbaceous cover (grass or crop) to each grid cell in quantities complementary to that of the grid cell’s fractional tree cover estimate (Eq. 9)

9 $${\mu }_{T}={\mu }_{w}+{\mu }_{h}\left(1-q\right)$$

where *μ*_*T*_ is the total biomass estimate of a grid cell, *μ*_*w*_ is the woody biomass estimate for the grid cell, *μ*_*h*_ is its herbaceous biomass estimate, and *q* is the MODIS fractional tree cover of the grid cell. Since MODIS tree cover estimates saturate at around 80%^64^, we linearly stretched values such that 80% was treated as complete tree cover (100%). Moreover, we acknowledge that percent cover can realistically exceed 100% when understory cover is considered but we were unable to reasonably determine the extent of underlying cover from satellite imagery. As such, our approach may underestimate the contribution of herbaceous C stocks in densely forested grid cells. The most likely herbaceous cover type was determined from the CCI Landcover 2010 map, which we aggregated into two “likely herbaceous cover” classes – grass or crop – based on the assumed likelihood of cropland in each CCI class (Online-only Table 1). However, due to inherent classification error in the native CCI Landcover map, when determining the herbaceous biomass contribution we weighted the relative allocation of crop and grass biomass to a given grid cell based on the probability of correct classification by the CCI map (i.e. “user’s accuracy”, Table 6) of the most probable herbaceous class (${p}_{i}$) such that *μ*_*h*_ can be further expressed as (Eq. 10)

10 $${\mu }_{h}={\mu }_{i}{p}_{i}+{\mu }_{j}\left(1-{p}_{i}\right)$$

where *μ*_*i*_ is the predicted biomass of the most probable herbaceous class, and *μ*_*j*_ is that of the less probable class.

**Table 6:** Area-weighted confusion matrix for “likely herbaceous” classes.

	Crop	Non-Crop	User’s Acc.
Crop	0.0314	0.0081	79%
Non-crop	0.0344	0.9261	96%
Prod. Acc.	48%	99%	**Overall**: 96%

Aggregated “likely herbaceous” classes were aggregated from the ESA CCI 2010 landcover map as described in Online-only Table 1. Class accuracies were taken from the ESA CCI matrix for the year 2010 as reported in Tables 4–5 in version 2.5 of the D3.4-PUG CCI Landcover Product User Guide^84^ and area-weighted following Olofsson *et al*.^44^. Area weighted user’s accuracies were used to propagate uncertainty associated with herbaceous biomass allocation.

The uncertainty of a grid cell’s total AGBC or BGBC estimate (${\sigma }_{T}$) was determined and mapped from that of its components (${\mu }_{w}\,{\rm{and}}\,{\mu }_{h}$) by summation in quadrature which can be simplified as (Eq. 11)

11 $${\sigma }_{T}=\sqrt{{\sigma }_{w}^{2}+{\left({\mu }_{h}q\sqrt{{\left(\frac{{\sigma }_{h}}{{\mu }_{h}}\right)}^{2}+{\left(\frac{{\sigma }_{q}}{q}\right)}^{2}}\right)}^{2}}$$

where ${\sigma }_{w}$ is the error of the grid cell’s estimated *μ*_*w*_, ${\sigma }_{h}$ is the error of its estimated *μ*_*h*_, and ${\sigma }_{q}$ is the error of its *q*. Here, ${\sigma }_{h}$ can be further decomposed and expressed as Eq. 12 to account for the accuracy weighted allocation procedure expressed previously (Eq. 10)

12 $${\sigma }_{h}=\sqrt{{\left({\mu }_{i}{p}_{i}\sqrt{{\left(\frac{{\sigma }_{i}}{{\mu }_{i}}\right)}^{2}+{\left(\frac{{\delta }_{i}}{{p}_{i}}\right)}^{2}}\right)}^{2}+{\left({\mu }_{j}\left(1-{p}_{i}\right)\sqrt{{\left(\frac{{\sigma }_{j}}{{\mu }_{j}}\right)}^{2}+{\left(\frac{{\delta }_{i}}{{p}_{i}}\right)}^{2}}\right)}^{2}}$$

where ${\sigma }_{i}$ is the error of the estimated biomass density of the most probable herbaceous class, ${\delta }_{i}$ is the estimated standard deviation of that class’s Bernoulli probability (*p*; Eq. 7), and ${\sigma }_{j}$ is the error of the estimated biomass density of the less probable herbaceous subclass.

Exceptions to the above schema were made in the tundra and boreal biomes – as delineated by the RESOLVE Ecoregions 2017 biome polygons^65^ – where thematic overlap was likely between the woody and tundra plant biomass maps. A separate set of decision rules (Fig. 3) was used to determine whether grid cells in these biomes were to be exclusively allocated the estimate of the tundra plant map or that of the fractional allocation procedure described above. In general, any land in these biomes identified as sparse landcover by the CCI landcover map (Online-only Table 1) was assigned the tundra vegetation estimate. In addition, lands north of 60° latitude with less than 10% tree cover or where the tundra AGBC estimate exceeded that of the woody AGBC estimate were also exclusively assigned the tundra vegetation estimate. Lands north of 60° latitude not meeting these criteria were assigned the woody value with the additional contribution of grass.

Subtle numerical artefacts emerged from the divergent methodologies employed north and south of 60°N latitude. These were eliminated by distance weighting grid cells within 1° of 60°N based on their linear proximity to 60°N and then averaging estimates such that values at or north of 61°N were exclusively based on the northern methodology, those at 60°N were the arithmetic average of the two methodologies and those at or south of 59°N were exclusively based on the southern methodology. This produced a seamless, globally harmonized product that integrates the best remotely sensed estimates of landcover-specific C density. Water bodies identified as class “210” of the CCI 2010 landcover map were then masked from our final products.

## Data Records

**Fig. 4:**
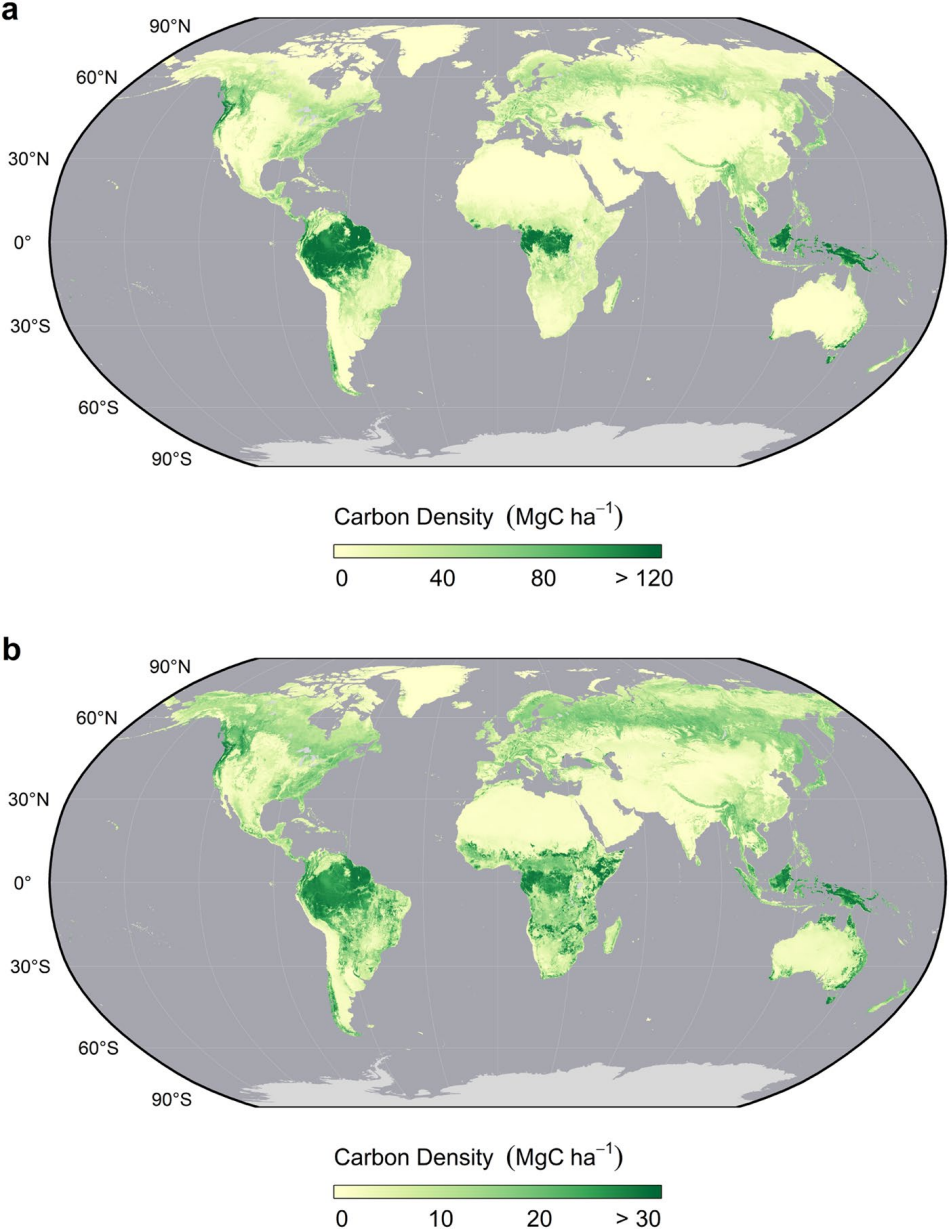
Globally harmonized maps of above and belowground living biomass carbon densities. (**a**) Aboveground biomass carbon density (AGBC) and (**b**) belowground biomass carbon density (BGBC) are shown separately. Maps have been aggregated to a 5 km spatial resolution and reprojected here for visualization.

**Fig. 5:**
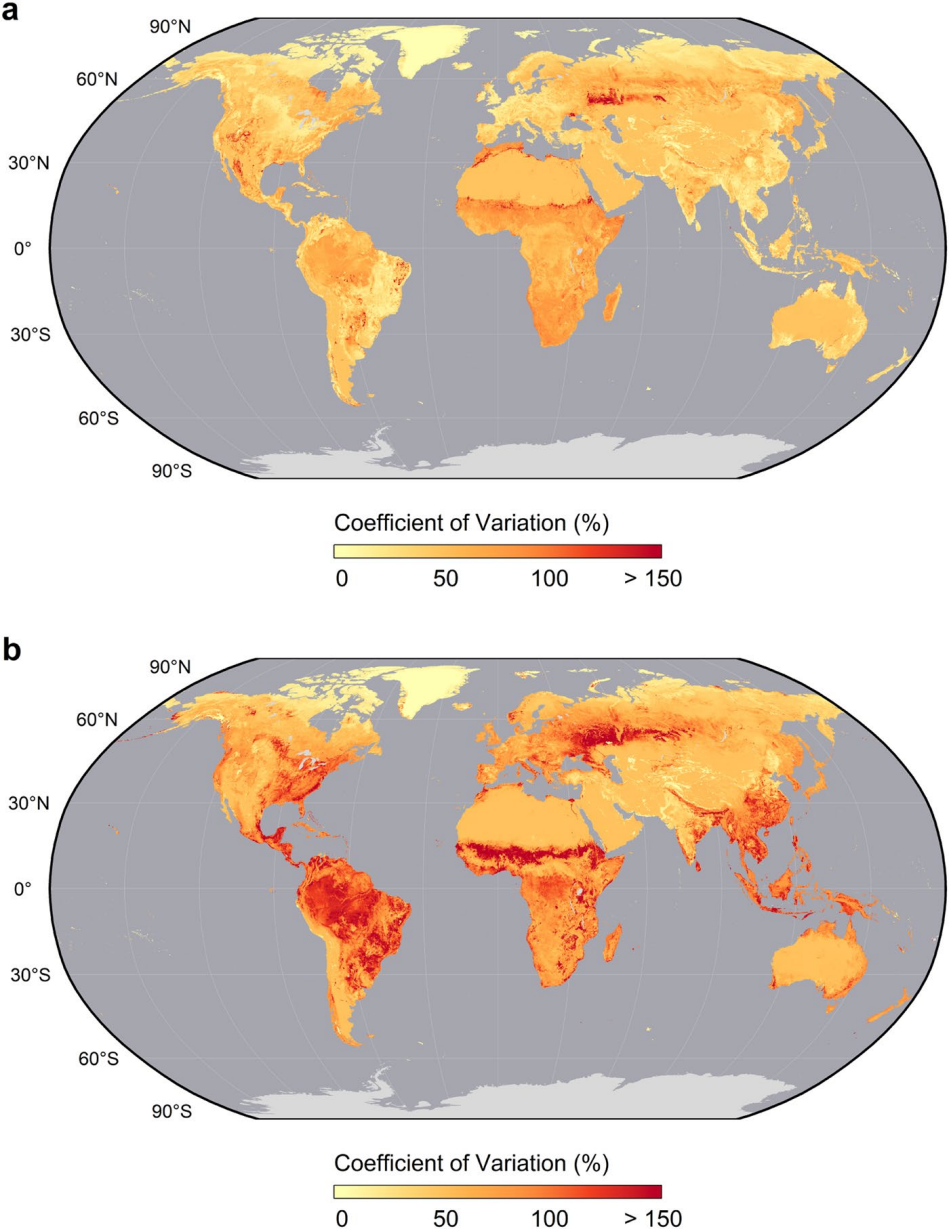
Uncertainty of grid cell level above and belowground biomass carbon density estimates. Uncertainty is shown here as the coefficient of variation (%; standard error layer divided by mean estimate layer) of estimated AGBC (**a**) and BGBC (**b**) densities after harmonization. Maps have been aggregated to a 5 km spatial resolution and projected for visualization.

Data layers (n = 4, Table 7) for the maps of AGBC and BGBC density (Fig. 4) as well as their associated uncertainty maps which represent the combined standard error of prediction (Fig. 5) are available as individual 16-bit integer rasters in GeoTiff format. All layers are natively in a WGS84 Mercator projection with a spatial resolution of approximately 300 m at the equator and match that of the ESA CCI Landcover Maps^37^. Raster values are in units megagrams C per hectare (MgC ha^−1^) and have been scaled by a factor of ten to reduce file size. These data are accessible through the Oak Ridge National Laboratory (ORNL) DAAC data repository (10.3334/ORNLDAAC/1763)^66^. In addition, updated and/or derived vegetation-specific layers that were used to create our harmonized 2010 maps are available as supplemental data on *figshare*^67^.

**Table 7:** Description of gridded data layers. Data layers should be multiplied by the scale factor to get raster values with units MgC ha^−1^.

Raster Layer	Description	Units	Scale Factor
agbc_2010.tif	Aboveground living biomass carbon stock density in 2010	MgC ha^−1^	0.1
bgbc_2010.tif	Belowground living biomass carbon stock density in 2010	MgC ha^−1^	0.1
agbc_2010_uncert.tif	Cumulative uncertainty (standard error) of aboveground living biomass carbon stock density in 2010 estimates	MgC ha^−1^	0.1
Bgbc_2010_uncert.tif	Cumulative uncertainty (standard error) of belowground living biomass carbon stock density in 2010 estimates	MgC ha^−1^	0.1

## Technical Validation

Our harmonized products rely almost exclusively upon maps and models that have been rigorously validated by their original producers and were often accompanied by constrained uncertainty estimates. Throughout our harmonization procedure, we strived to conserve the validity of each of these products by minimizing the introduction of additional error and by tracking any introductions, as described above, such that the final error layers represent the cumulative uncertainty of the inputs used. Ground truth AGB and BGB data are almost always collected for individual landcover types. Consequently, we are unable to directly assess the validity of our integrated estimates beyond their relationships to individual landcover-specific estimates and the extents to which they were modified from their original, previously-validated form prior to and during our harmonization procedure.

### Modifications to independent biomass layers

**Fig. 6:**
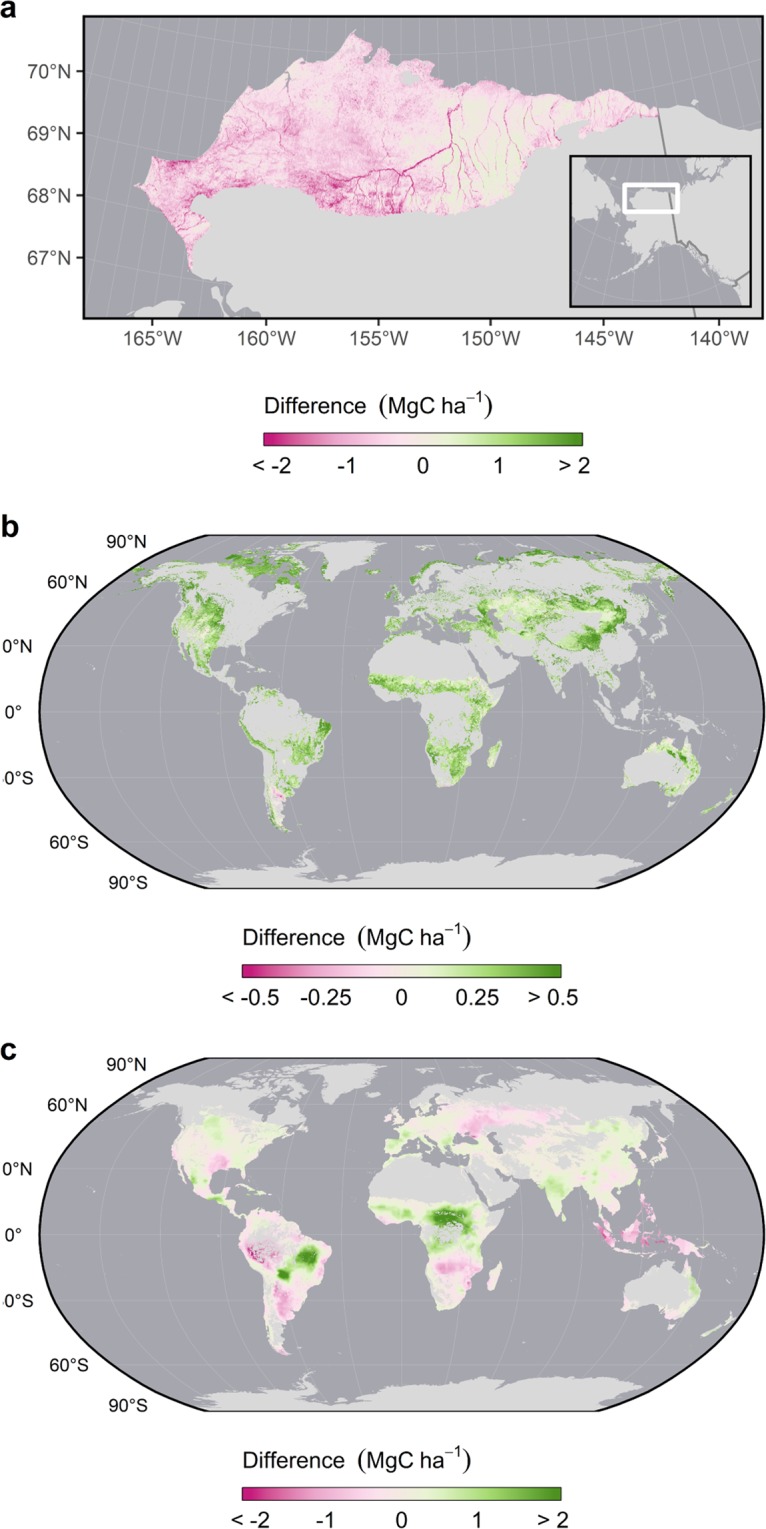
Differences between landcover-specific AGBC estimates from the original published maps and the modified versions used as inputs to create the 2010 harmonized global maps. Tundra vegetation AGBC (**a**) is compared to the Landsat-based map of Berner *et al*.^45^ for the north slope of Alaska after converting it to units MgC ha^−1^. Here, the comparison map was subsequently aggregated to a 1 km resolution and reprojected for visualization. Grassland AGBC (**b**) is compared to the AVHRR-based map of Xia *et al*.^19^ which represents the average estimate between 1982–2006. For visualization, the map was aggregated to a 5 km resolution and subsequently reprojected after being masked to MODIS IGBP grasslands in the year 2006^85^ following Xia *et al*.^19^. As such, this map does not necessarily represent the spatial distribution of grid cells in which grassland estimates were used. Cropland AGBC (**c**) is compared to the original circa 2000 estimates to assess the effects of the 2000-to-2010 correction. The map is masked to the native extent of the combined yield maps and aggregated to a 5 km resolution for visualization. For all maps, negative values indicate that our circa 2010 estimates are lower than those of the earlier maps while positive values indicate higher estimates.

Temporal and spatial updates made to existing landcover-specific maps of non-tree AGB resulted in relatively small changes to their predictions. For example, we used numerically calibrated MODIS imagery to extend the Landsat-based tundra plant AGB model beyond its native extent (the North Slope of Alaska) to the pan-Arctic region since neither a comparable model nor a consistent Landsat time series were available for this extent. We assessed the effects of these assumptions by comparing our predictions for the North Slope with those of the original map^18^ (Fig. 6a). Both positive and negative discrepancies exist between ours and the original, though these rarely exceed ±2 MgC ha^−1^ and no discernibly systematic bias was evident.

Our updated map of grassland biomass carbon in the year 2010 was similarly made by applying the original AVHRR-based model to calibrated MODIS imagery. This too resulted in only subtle changes to the original biomass map (Fig. 6b) that were rarely in excess of 0.5 MgC ha^−1^. In most areas, our estimates were higher than those of Xia *et al*.^19^ who mapped the mean AGBC density between 1986 and 2006. Most of these elevated estimates corresponded with areas in which significant NDVI increases (“greening”) have been reported while notably lower estimates in the Argentine Monte and Patagonian steppe biomes of southern South America, likewise, correspond with areas of reported “browning”^68,69^. Both greening and browning trends are well documented phenomena and have been linked to climatic changes^70^. Moreover, we further compared AGBC estimates from both the original Xia *et al*.^19^ map and our 2010 update to AGBC field measurements coordinated by the Nutrient Network that were collected from 48 sites around the world between 2007 and 2009^49^. The RMSE (0.68 MgC ha^−1^) of our updated map was 10% less that of the Xia *et al*. map for sites with less than 40% tree cover. Likewise, our 2010 estimates were virtually unbiased (bias = −0.01 MgC ha^−1^) in comparison to the Xia map (bias = 0.25 MgC ha^−1^). While still noisy, these results suggest that our temporal update improved the overall accuracy of estimated grassland AGBC.

Finally, cropland biomass carbon maps were also updated from their native epoch (2000) to 2010 using pixel-wise rates of MODIS ANPP change over a ten-year period. While MODIS ANPP may be a poor snapshot of crop biomass in a single year, we assumed that its relative change over time reflects real physiological shifts affecting the cropland C cycle. This correction also resulted in only small differences that rarely exceeded ±2 MgC ha^−1^ and that, spatially, correspond well with observed declines in the yields of select crops that have been linked to climate change^71,72^ (Fig. 6c). Nonetheless, updated global yield maps comparable to those available for 2000 would greatly improve our understanding of the interactions between climate change, crop yields, and C dynamics.

### Belowground biomass estimates

Belowground biomass is notoriously difficult to measure, model, and also to validate. We accounted for the reported uncertainty of nearly every variable considered when estimating belowground biomass and pixel-level uncertainty, but we were unable to perform an independent validation of our harmonized estimates at the pixel level due to a paucity of globally consistent field data. To complete such a task, a globally orchestrated effort to collect more BGB samples data across all vegetation types is needed.

Given this lack of data, we instead compared the estimated uncertainty of our BGBC maps to that of our AGBC estimates to infer the sources of any divergence (Fig. 5). As expected, our cumulative BGBC uncertainty layer generally reveals greater overall uncertainty than our AGBC estimates, with BGBC uncertainty roughly twice that of AGBC throughout most of the globe. The highest absolute uncertainty was found in biomass rich forests. Arid woodlands, especially those of the Sahel and eastern Namibia, generally had the greatest relative BGBC uncertainty, though their absolute uncertainty was quite small (generally less than 3 MgC ha^−1^). Here, biomass estimates of sparse woody vegetation were primarily responsible for heightened relative uncertainty. High relative and absolute BGBC uncertainty were also associated with predictions in select mountainous forests (e.g. east central Chile) as well as forested areas in and around cities. These patterns were largely driven by AGB uncertainty in the GlobBiomass product.

### Biomass harmonization

The GlobBiomass global woody AGB map produced by Santoro *et al*.^30^ comprises the backbone of our integrated products and, with few exceptions, remains largely unchanged in our final AGBC map. The native version of the GlobBiomass map is accompanied by an error layer describing the uncertainty of each pixel’s biomass estimate and this too forms the core of our integrated uncertainty layers. In areas with tree cover, the global average error of GlobBiomass estimates is 39 Mg ha^−1^ or 50% with greater relative uncertainty in densely forested areas, along the margins of forested expanses like farm fields and cities, and in similar areas with sparse tree cover.

**Fig. 7:**
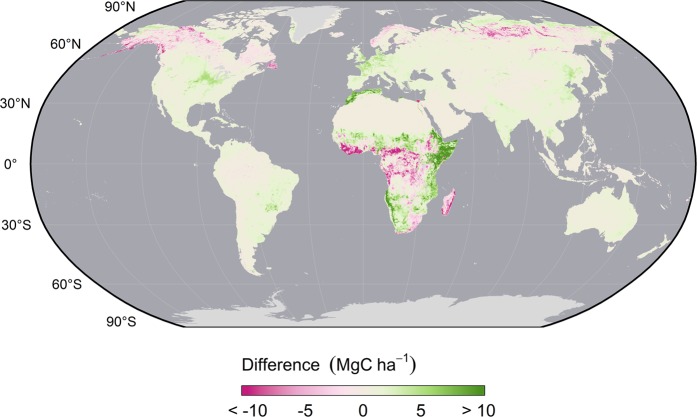
Differences between the final harmonized AGBC map and GlobBiomass AGBC. GlobBiomass AGB was aggregated to a 300 m spatial resolution and converted to C density prior to comparison. Negative values indicate areas where the new map reports lower values than GlobBiomass while positive value denote higher estimates.

Adding additional grass or crop biomass in complementary proportion to a grid cell’s tree cover often did not exceed the estimated error of the original GlobBiomass map (Fig. 7). Grid cells exceeding GlobBiomass’s native uncertainty comprise less than 40% of its total extent. Exceptions were primarily found in grassland and cropland dominated regions where tree cover was generally sparse, and, consequently, the herbaceous biomass contribution was relatively high. Even so, the absolute magnitude of these additions remains somewhat small (less than 2.3 MgC ha^−1^ for grassland and 15 MgC ha^−1^ for cropland).

Larger deviations from GlobBiomass were also present in areas of both dryland Africa and the Arctic tundra biome, where we used independent layers to estimate woody biomass. In African drylands, GlobBiomass likely underestimates woody biomass by adopting the conservative FAO definition (DBH > 10 cm), which implicitly omits the relatively small trees and shrubs that are common to the region. The Bouvet map of Africa that we used to supplement these estimates is not bound by this constraint, was developed from region-specific data, and predicts substantially higher AGB density throughout much of its extent with comparatively high accuracy (RMSE = 17.1 Mg ha^−1^)^35^.

GlobBiomass also included sporadic biomass estimates throughout much of the Arctic tundra biome. Trees are generally scarce throughout this biome, which is instead dominated by dwarf shrubs and herbaceous forbs and graminoids, so given GlobBiomass’s adherence to FAO guidelines, its predictions here may be spurious. We thus prioritized the estimates of the independent model developed specifically to collectively predict biomass of both woody and herbaceous tundra vegetation. These estimates were generally higher than GlobBiomass but agreed well with independent validation data from North America (RMSE = 2.9 Mg ha^−1^)^18^.

### Comparison with the IPCC Tier-1 global biomass carbon map

While far from a perfect comparison, the only other map to comprehensively report global biomass carbon density for all landcover types is the IPCC Tier-1 map for the year 2000 by Ruesch and Gibbs^28^. As previously described, this map was produced using an entirely different method (“stratify and multiply”) and distinct data sources^23^ and represents an earlier epoch. However, the map is widely used for myriad applications, and it may thus be informative to assess notable differences between it and our new products.

Ruesch and Gibbs^28^ report total living C stocks of 345 petagrams (PgC) in AGBC and 133 PgC in BGBC for a total of 478 PgC, globally. Our estimates are lower at 287 PgC and 122 PgC in global AGBC and BGBC, respectively, for a total of 409 PgC in living global vegetation biomass. Herbaceous biomass in our maps comprised 9.1 and 28.3 PgC of total AGBC and BGBC, respectively. Half of all herbaceous AGBC (4.5 PgC) and roughly 6% of all herbaceous BGBC (1.7 PgC) was found in croplands. Moreover, we mapped 22.3 and 6.1 PgC, respectively, in the AGB and BGB of trees located within the cropland extent. These trees constituted roughly 7% of all global biomass C and are likely overlooked by both the Ruesch and Gibbs map^28^ and by remotely sensed forest C maps that are masked to forested areas. Zomer *et al*.^17^ first highlighted this potential discrepancy in the Ruesch and Gibbs map^28^ when they produced a remarkably similar estimate of 34.2 Pg of overlooked C in cropland trees using Tier-1 defaults. However, their estimates were assumed to be in addition to the 474 PgC originally mapped by Ruesch and Gibbs^28^. Here, we suggest that the 28.4 PgC we mapped in cropland trees is already factored into our 409 PgC total.

**Fig. 8:**
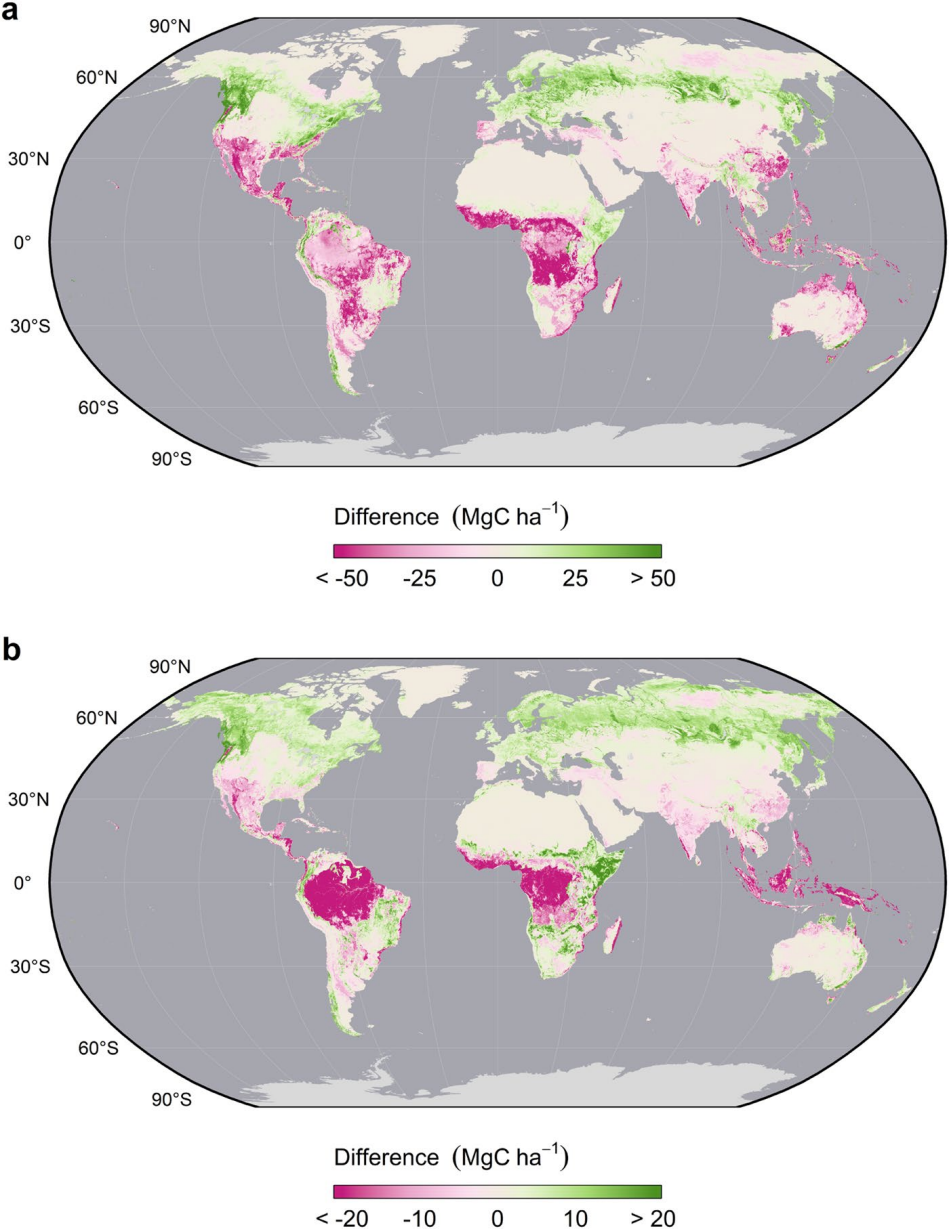
Differences between the 2010 harmonized global maps of above and belowground biomass carbon density and those of the IPCC Tier-1 product by Ruesch and Gibbs for 2000. Comparisons of AGBC (**a**) and BGBC (**b**) maps are shown separately. Negative values indicate that the circa 2010 estimates are comparatively lower while positive values indicate higher estimates.

Our AGBC product predicts substantially less biomass C than Ruesch and Gibbs^28^ throughout most of the pantropical region and, to a lesser extent, southern temperate forests (Fig. 8a). This pattern has been noted by others comparing the Ruesch and Gibbs map^28^ to other satellite-based biomass maps^73^ and may suggest that the IPCC default values used to create it^23^ are spatially biased. In addition, well-defined areas of high disagreement emerge in Africa that directly correspond with the FAO boundaries of the “tropical moist deciduous forest” ecofloristic zone and suggest that this area, in particular, may merit critical review. Moreover, the opposite pattern is observed in this same ecofloristic zone throughout South America. Our map also predicts greater AGBC throughout much of the boreal forest as well as in African shrublands and the steppes of South America.

We observed similar, though less pronounced discrepancies, when comparing BGBC maps (Fig. 8b). Notably, our map predicts substantially more BGBC throughout the tundra biome – a previously underappreciated C stock that has recently risen to prominance^74^ – the boreal forest, African shrublands and most of South America and Australia. However, we predict less BGBC in nearly all rainforests (Temperate and Tropical). These differences and their distinct spatial patterns correspond with the vegetation strata used to make the IPCC Tier-1 map^28^ and suggest that the accuracy of the “stratify and multiply” method depends heavily upon the quality of the referenced and spatial data considered. Inaccuracies in these data may, in turn, lead to false geographies. Integrating, continuous spatial estimates that better capture local and regional variation, as we have done, may thus greatly improve our understanding of global carbon geographies and their role in the earth system.

#### Congruence with IPCC Tier-2 and Tier-3 nationally reported woody carbon stocks

**Fig. 9:**
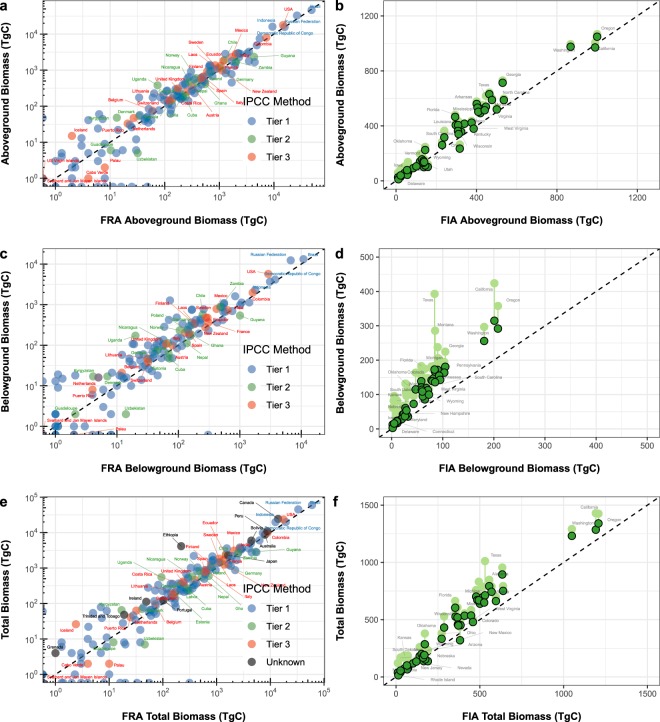
Comparison of woody biomass density estimates to corresponding estimates of the FAO’s FRA and the USFS’s FIA. National woody AGBC totals derived from the woody components of our harmonized maps are compared to national totals reported in the 2015 FRA^62^ (**a**) in relation to the IPCC inventory methodology used by each country. Likewise, we derived woody AGBC totals for US states and compared them to the corresponding totals reported by the 2014 FIA^75^ (**b**), a Tier-3 inventory. We also show the additional effect of considering non-woody C – as is reported in our harmonized maps – in light green. Similar comparisons were made between our woody BGBC estimates and the corresponding estimates of both the FRA (**c**) and FIA (**d**). We further summed our woody AGBC and BGBC estimates and compared them to the total woody C stocks reported by both the FRA (**e**) and FIA (**f**).

The error and variance between our woody biomass estimates – when aggregated to the country level – and comparable totals reported in the FRA were less for comparisons made against FRA estimates generated using higher tier IPCC methodologies than for those based on Tier-1 approaches (Fig. 9). Across the board for AGBC, BGBC, and total C comparisons, the relative RMSE (RMSE_CV_) of our estimates, when compared to estimates generated using high tier methods, was roughly half of that obtained from comparisons with Tier-1 estimates (Table 8). Likewise, the coefficient of determination (R^2^) was greatest for comparisons with Tier-3 estimates. For each pool-specific comparison (AGBC, BGBC, and total C), the slopes of the relationships between Tier-1, 2, and 3 estimates were neither significantly different from a 1:1 relationship nor from one another (p > 0.05; ANCOVA). Combined, these results suggest that our maps lead to C stock estimates congruent with those attained from independent, higher-tier reporting methodologies.

**Table 8:** Statistical comparison of woody biomass carbon totals derived from the 2010 harmonized maps and those reported by the FRA in relation to the IPCC inventory methodology used.

Reporting Method	N	Slope	R^2^	RMSE_CV_ (%)
***Aboveground Woody Biomass Carbon***				
Tier-1	136	0.983	0.884	23.6
Tier-2	18	0.949	0.819	13.0
Tier-3	25	0.999	0.963	10.5
“High Tier” (2 & 3)	43	0.987	0.931	11.7
***Belowground Woody Biomass Carbon***				
Tier-1	135	1.016	0.856	32.4
Tier-2	18	0.928	0.766	20.6
Tier-3	23	1.000	0.944	16.6
“High Tier” (2 & 3)	41	0.981	0.895	18.5
***Total Woody Biomass Carbon***				
Tier-1	136	0.983	0.853	26.4
Tier-2	18	0.946	0.816	13.2
Tier-3	25	0.997	0.960	11.2
“High Tier” (2 & 3)	43	0.984	0.927	12.1

Statistics for AGBC, BGBC, and total C correspond to relationships depicted in Fig. 9a,c,f, respectively.

To explore this association at a finer regional scale, we also compared our woody C estimates to the United States Forest Service’s Forest Inventory Analysis^75^ (FIA) and found similarly strong congruence for AGBC and Total C stocks but subtle overestimates for BGBC (Fig. 9). The FIA is a Tier-3 inventory of woody forest biomass C stocks that is based on extensive and statistically rigorous field sampling and subsequent upscaling, We used data available at the state level for the year 2014 – again, the only year in which we could obtain data partitioned by AGBC and BGBC. Like our FRA comparison, we found a tight relationship between our woody AGBC totals and those reported by the FIA (Fig. 9b; RMSE_CV_ = 25.7%, R^2^ = 0.960, slope = 1.10, n = 48). Our woody BGBC estimates, though, were systematically greater than those reported by the FIA (Fig. 9d; RMSE_CV_ = 86.4%, R^2^ = 0.95, slope = 1.51, n = 48). This trend has been noted by others^27^ and suggests that the global model that we used to estimate woody BGBC may not be appropriate for some finer scale applications as is foretold by the elevated uncertainty reported in our corresponding uncertainty layer (Fig. 5b). Our total woody C (AGBC + BGBC) estimates (Fig. 9f), however, agreed well with the FIA (RMSE_CV_ = 34.1%, R^2^ = 0.961, slope = 1.17, n = 48) and thus reflect the outsized contribution of AGBC to the total woody C stock. When the contribution of herbaceous C stocks is further added to these comparisons, our stock estimates intuitively increase in rough proportion to a state’s proportional extent of herbaceous cover. The effect of this addition is particularly pronounced for BGBC estimates due to the large root-to-shoot ratios of grassland vegetation.

The relative congruence of our results with higher-tier stock estimates suggests that our maps could be used to facilitate broader adoption of higher-tier methods among countries currently lacking the requisite data and those seeking to better account for C in non-woody biomass. This congruence spans a comprehensive range of biophysical conditions and spatial scales ranging from small states to large nations. Moreover, a recent study suggests that the fidelity of the underlying GlobBiomass AGB map may extend to even finer scales^31^. While our BGBC estimates may differ from some fine-scale estimates (Fig. 9d), their tight agreement with high tier BGBC totals at the national level (Fig. 9c) suggests that they may still be well suited for many national-scale C inventories – especially for countries lacking requisite high tier data. Use of our maps is unlikely to introduce error in excess of that currently implicit in Tier-1 estimates. Credence, though, should be given to the associated uncertainty estimates. To facilitate wider adoption of higher-tier methodologies, our maps could be used to derive new, region-specific default values for use in Tier-2 frameworks^76^ or to either represent or calibrate 2010 baseline conditions in Tier-3 frameworks. In so doing, inventories and studies alike could more accurately account for the nuanced global geographies of biomass C.

## Usage Notes

These maps are intended for global applications in which continuous spatial estimates of live AGBC and/or BGBC density are needed that span a broad range of vegetation types and/or require estimates circa 2010. They are loosely based upon and share the spatial resolution of the ESA CCI Landcover 2010 map^37^, which can be used to extract landcover specific C totals. However, our products notably do not account for C stored in non-living C pools like litter or coarse woody debris, nor soil organic matter, though these both represent large, additional ecosystem C stocks^77–79^. Our maps are explicitly intended for global scale applications seeking to consider C in the collective living biomass of *multiple* vegetation types. For global scale applications focused exclusively on the C stocks of a single vegetation type, we strongly encourage users to instead use the respective input map or model referenced in Table 1 to avoid potential errors that may have been introduced by our harmonization procedure. For AGB applications over smaller extents, users should further consider whether locally specific products are available. If such maps are not available and our maps are considered instead, credence should be given to their pixel-level uncertainty estimates. As mentioned above, the biomass of shrublands was only explicitly accounted for in Africa and the Arctic tundra, since neither broad-scale maps nor models generalizable to other areas were available in the existing literature. As such, we caution against the use of our maps outside of these areas when shrubland biomass is of particular interest or importance. Moreover, in contrast to the estimates for all other vegetation types considered, which we upscaled to a 300 m resolution, cropland C estimates were largely based on relatively coarse 8 km resolution data that were downscaled using bilinear resampling to achieve a 300 m spatial resolution. As such, these estimates may not adequately capture the underlying finer-scale spatial variation and should be interpreted with that in mind. Likewise, we reiterate that some BGBC estimates may differ from locally derived Tier-3 estimates, and attention should thus be given to our reported pixel-level uncertainty for all applications. Finally, our maps should not be used in comparison with the IPCC Tier-1 map of Ruesch and Gibbs (2008) to detect biomass change between the two study periods due to significant methodological differences between these products.

## Data Availability

Cropland biomass maps were created in the R statistical computing environment^80^. All other coding was done in Google Earth Engine^81^ (GEE), wherein our workflow consisted of 18 interconnected scripts. All code can be found on GitHub (https://github.com/sethspawn/globalBiomassC) and permanently archived by Zenodo^82^.

## Online-only Tables

**Online-only Table 1:** Reclassification table of the CCI Landcover 2010 map.

CCI Code	CCI Class	Woody AGB Source in Africa	Method used for Woody BGB Mapping	Forest Phylogeny	Likely Herb. Class	Sparse Cover
0	No Data	Bouvet *et al*.^35^	Reich *et al*.^25^	Mixed	Grass	—
10	Cropland, rainfed	Bouvet *et al*.^35^	Reich *et al*.^25^	Mixed	Crop	—
11	Cropland, rainfed	Bouvet *et al*.^35^	Reich *et al*.^25^	Mixed	Crop	—
12	Cropland, rainfed	Bouvet *et al*.^35^	Reich *et al*.^25^	Mixed	Crop	—
20	Cropland, irrigated or post‐flooding	Bouvet *et al*.^35^	Reich *et al*.^25^	Mixed	Crop	—
30	Mosaic cropland (>50%)/natural vegetation (tree, shrub, herbaceous cover) (<50%)	Bouvet *et al*.^35^	Reich *et al*.^25^	Mixed	Crop	—
40	Mosaic natural vegetation (tree, shrub, herbaceous cover) (>50%)/cropland (<50%)	Bouvet *et al*.^35^	Reich *et al*.^25^	Mixed	Crop	—
50	Tree cover, broadleaved, evergreen, closed to open (>15%)	Santoro *et al*.^30^	Reich *et al*.^25^	Gymnosperm	Grass	—
60	Tree cover, broadleaved, deciduous, closed to open (>15%)	Santoro *et al*.^30^	Reich *et al*.^25^	Gymnosperm	Grass	—
61	Tree cover, broadleaved, deciduous, closed (>40%)	Santoro *et al*.^30^	Reich *et al*.^25^	Gymnosperm	Grass	—
62	Tree cover, broadleaved, deciduous, open (15‐40%)	Bouvet *et al*.^35^	Mokany *et al*.^22^ (Savannah)	Mixed	Grass	—
70	Tree cover, needleleaved, evergreen, closed to open (>15%)	Santoro *et al*.^30^	Reich *et al*.^25^	Angiosperm	Grass	—
71	Tree cover, needleleaved, evergreen, closed (>40%)	Santoro *et al*.^30^	Reich *et al*.^25^	Angiosperm	Grass	—
72	Tree cover, needleleaved, evergreen, open (15‐40%)	Bouvet *et al*.^35^	Reich *et al*.^25^	Angiosperm	Grass	—
80	Tree cover, needleleaved, deciduous, closed to open (>15%)	Santoro *et al*.^30^	Reich *et al*.^25^	Angiosperm	Grass	—
81	Tree cover, needleleaved, deciduous, closed (>40%)	Santoro *et al*.^30^	Reich *et al*.^25^	Angiosperm	Grass	—
82	Tree cover, needleleaved, deciduous, open (15‐40%)	Bouvet *et al*.^35^	Reich *et al*.^25^	Angiosperm	Grass	—
90	Tree cover, mixed leaf type (broadleaved and needleleaved)	Santoro *et al*.^30^	Reich *et al*.^25^	Mixed	Grass	—
100	Mosaic tree and shrub (>50%)/herbaceous cover (<50%)	Bouvet *et al*.^35^	Reich *et al*.^25^	Mixed	Grass	—
110	Mosaic herbaceous cover (>50%)/tree and shrub (<50%)	Bouvet *et al*.^35^	Reich *et al*.^25^	Mixed	Grass	—
120	Shrubland	Bouvet *et al*.^35^	Mokany *et al*.^22^ (Shrub)	Mixed	Grass	Sparse
121	Evergreen shrubland	Bouvet *et al*.^35^	Mokany *et al*.^22^ (Shrub)	Mixed	Grass	Sparse
122	Deciduous shrubland	Bouvet *et al*.^35^	Mokany *et al*.^22^ (Shrub)	Mixed	Grass	Sparse
130	Grassland	Bouvet *et al*.^35^	Reich *et al*.^25^	Mixed	Grass	Sparse
140	Lichens and mosses	Bouvet *et al*.^35^	Reich *et al*.^25^	Mixed	Grass	Sparse
150	Sparse vegetation (tree, shrub, herbaceous cover) (<15%)	Bouvet *et al*.^35^	Reich *et al*.^25^	Mixed	Grass	Sparse
151	Sparse tree (<15%)	Bouvet *et al*.^35^	Mokany *et al*.^22^ (Savannah)	Mixed	Grass	Sparse
152	Sparse shrub (<15%)	Bouvet *et al*.^35^	Mokany *et al*.^22^ (Shrub)	Mixed	Grass	Sparse
153	Sparse herbaceous cover (<15%)	Bouvet *et al*.^35^	Mokany *et al*.^22^ (Savannah)	Mixed	Grass	Sparse
160	Tree cover, flooded, fresh or brakish water	Santoro *et al*.^30^	Reich *et al*.^25^	Mixed	Grass	—
170	Tree cover, flooded, saline water	Santoro *et al*.^30^	Reich *et al*.^25^	Mixed	Grass	—
180	Shrub or herbaceous cover, flooded, fresh/saline/brakish water	Santoro *et al*.^30^	Mokany *et al*.^22^ (Shrub)	Mixed	Grass	—
190	Urban areas	Bouvet *et al*.^35^	Reich *et al*.^25^	Mixed	Grass	—
200	Bare areas	Bouvet *et al*.^35^	Reich *et al*.^25^	Mixed	Grass	Sparse
201	Consolidated bare areas	Bouvet *et al*.^35^	Reich *et al*.^25^	Mixed	Grass	Sparse
202	Unconsolidated bare areas	Bouvet *et al*.^35^	Reich *et al*.^25^	Mixed	Grass	Sparse
210	Water bodies	Bouvet *et al*.^35^	Reich *et al*.^25^	Mixed	Grass	—
220	Permanent snow and ice	Bouvet *et al*.^35^	Reich *et al*.^25^	Mixed	Grass	—

CCI^37^ classes were variously aggregated to (i) determine the source of woody AGB estimates in Africa, (ii) determine the method used for mapping woody BGBC, (iii) determining the phylogeny of woody AGB for BGB mapping and applying biomass C fractions, (iv) determining a grid cell’s “likely herbaceous” class for map harmonization, and (v) identify areas of “sparse cover” for map harmonization in northern areas.

**Online-only Table 2:** Crop-specific harvest and physiological parameters used to convert gridded yields to AGBC and BGBC.

Monfreda^20^ Crop ID	Crop Description	Commodity Class	Harvest Index	Root:Shoot	Harvested Dry Matter Fraction	Harvested Dry Matter Carbon Fraction^21^	Secondary Source
alfalfa	Alfalfa	Forage	0.95^86^	0.87^87^	0.35^21^	0.44	Wolf *et al*. 2015^21^
bambara	Bambara beans	Pulses	0.40^87^	0.07^87^	0.91^87^	0.46	Wolf *et al*. 2015^21^
barley	Barley	Cereals	0.46^88–91^	0.11^92^	0.87^93^	0.46	Wolf *et al*. 2015^21^
bean	Beans, dry	Pulses	0.46^87^	0.08^87^	0.84^94^	0.46	Wolf *et al*. 2015^21^
beetfor	Beets for fodder	Forage	0.95^86^	0.43^87^	0.15^87^	0.41	Wolf *et al*. 2015^21^
broadbean	Broad beans, dry	Pulses	0.46^87^	0.08^87^	0.84^94^	0.46	Wolf *et al*. 2015^21^
buckwheat	Buckwheat	Cereals	0.43^91,95,96^	0.10^91^	0.87^96^	0.46	Wolf *et al*. 2015^21^
cabbage	Cabbage for fodder	Forage	0.80^86^	0.15^97^	0.08^98^	0.41	Wolf *et al*. 2015^21^
canaryseed	Canary seed	Cereals	0.40^99^	0.25^100^	0.88^99^	0.46	Monfreda *et al*. 2008^20^
carrotfor	Carrots for fodder	Forages	0.95^101^	0.15^97^	0.13^98^	0.41	Wolf *et al*. 2015^21^
cassava	Cassava	Roots & Tubers	0.50^102^	0.15^97^	0.88^103^	0.44	Wolf *et al*. 2015^21^
castor	Castor beans	Oil Crops	0.52^99^	0.25^100^	0.73^99^	0.60	Monfreda *et al*. 2008^20^
cerealnes	Cereals, other	Cereals	0.40^99^	0.25^100^	0.88^99^	0.46	Monfreda *et al*. 2008^20^
chickpea	Chickpeas	Pulses	0.46^87^	0.08^87^	0.87^21^	0.46	Wolf *et al*. 2015^21^
clover	Clover	Forage	0.95^86^	1.10^104^	0.35^21^	0.44	Wolf *et al*. 2015^21^
coir	Coir	Fiber	0.28^99^	0.25^100^	0.80^99^	0.49	Monfreda *et al*. 2008^20^
cotton	Cotton	Fiber	0.40^87^	0.17^87^	0.92^87^	0.54	Wolf *et al*. 2015^21^
cowpea	Cow peas, dry	Pulses	0.45^21^	0.08^21^	0.84^94^	0.46	Wolf *et al*. 2015^21^
fibrenes	Fiber crops, other	Fiber	0.28^99^	0.25^100^	0.80^99^	0.49	Monfreda *et al*. 2008^20^
flax	Flax fiber and tow	Fiber	0.28^99^	0.25^100^	0.80^99^	0.49	Monfreda *et al*. 2008^20^
fonio	Fonio	Cereal	0.25^104^	0.11^92^	0.89^21^	0.46	Wolf *et al*. 2015^21^
fornes	Forage products, other	Forage	1.00^20^	0.54^100^	0.20^105^	0.43	Monfreda *et al*. 2008^20^
grassnes	Grasses, other	Forage	0.95^86^	1.81^106^	0.35^21^	0.44	Wolf *et al*. 2015^21^
groundnut	Groundnuts in shell	Oil Crops	0.40^87^	0.07^87^	0.91^87^	0.60	Wolf *et al*. 2015^21^
hemp	Hemp fiber and tow	Fiber	0.28^99^	0.25^100^	0.80^99^	0.49	Monfreda *et al*. 2008^20^
hempseed	Hempseed	Oil Crops	0.18^16^	0.15^86^	0.91^107^	0.62	Wolf *et al*. 2015^21^
jute	Jute	Fiber	0.30^108^	0.10^109^	0.92^87^	0.44	Wolf *et al*. 2015^21^
jutelikefiber	Jute-like fibers	Fiber	0.30^108^	0.10^109^	0.92^87^	0.49	Wolf *et al*. 2015^21^
legumenes	Legumes, other	Forage	0.95^86^	1.10^106^	0.35^21^	0.44	Wolf *et al*. 2015^21^
lentil	Lentils	Pulses	0.61^101^	0.15^97^	0.84^94^	0.46	Wolf *et al*. 2015^21^
linseed	Linseed	Oil Crops	0.26^110^	0.15^97^	0.92^93^	0.62	Wolf *et al*. 2015^21^
lupin	Lupins	Pulses	0.41^111^	0.18^112^	0.89^113^	0.46	Monfreda *et al*. 2008^20^
maize	Maize	Cereals	0.53^87^	0.18^87^	0.86^93^	0.46	Wolf *et al*. 2015^21^
maizefor	Maize for forage and silage	Forage	0.95^86^	0.18^87^	0.35^21^	0.44	Wolf *et al*. 2015^21^
millet	Millet	Cereals	0.45^114^	0.25^115,116^	0.89^117^	0.46	Wolf *et al*. 2015^21^
mixedgrain	Mixed grain	Cereals	0.40^99^	0.25^100^	0.88^99^	0.46	Monfreda *et al*. 2008^20^
mixedgrass	Mixed grasses and legumes	Forage	1.00^20^	0.54^100^	0.20^105^	0.43	Monfreda *et al*. 2008^20^
mustard	Mustard seed	Oil Crops	0.30^101^	0.15^97^	0.92^21^	0.62	Wolf *et al*. 2015^21^
oats	Oats	Cereals	0.52^87^	0.40^87^	0.87^93^	0.46	Wolf *et al*. 2015^21^
oilseedfor	Green oilseeds for fodder	Forage	0.52^99^	0.25^100^	0.73^99^	0.43	Monfreda *et al*. 2008^20^
oilseednes	Oilseeds, other	Oil Crops	0.52^99^	0.25^100^	0.73^99^	0.60	Monfreda *et al*. 2008^20^
pea	Peas, dry	Pulses	0.30^101^	0.08^97^	0.87^97^	0.46	Wolf *et al*. 2015^21^
pigeonpea	Pigeon peas	Pulses	0.30^118^	0.08^97^	0.87^119^	0.46	Wolf *et al*. 2015^21^
popcorn	Pop corn	Cereals	0.53^87^	0.18^87^	0.86^93^	0.46	Wolf *et al*. 2015^21^
poppy	Poppy seed	Oil Crops	0.52^99^	0.25^100^	0.73^99^	0.60	Monfreda *et al*. 2008^20^
potato	Potatoes	Roots & Tubers	0.50^87^	0.07^87^	0.20^87^	0.41	Wolf *et al*. 2015^21^
pulsenes	Pulses, other	Pulses	0.49^99^	0.18^112^	0.90^112^	0.46	Monfreda *et al*. 2008^20^
quinoa	Quinoa	Cereals	0.28^91,95^	0.12^91^	0.87^117^	0.46	Wolf *et al*. 2015^21^
rapeseed	Rapeseed	Oil Crops	0.30^101^	0.15^97^	0.93^21^	0.62	Wolf *et al*. 2015^21^
rice	Rice	Cereals	0.42^89,120–123^	0.22^124^	0.91^87^	0.46	Wolf *et al*. 2015^21^
rootnes	Roots and tubers, other	Roots & Tubers	0.40^99^	0.25^100^	0.20^99^	0.42	Monfreda *et al*. 2008^20^
rye	Rye	Cereals	0.50^87^	0.14^125,126^	0.90^87^	0.46	Wolf *et al*. 2015^21^
ryefor	Rye grass for forage and silage	Forage	0.95^86^	1.50^106^	0.35^21^	0.44	Wolf *et al*. 2015^21^
safflower	Safflower seed	Oil Crops	0.20^127^	0.10^128^	0.92^129^	0.62	Wolf *et al*. 2015^21^
sesame	Sesame seed	Oil Crops	0.27^89^	0.15^97^	0.95^97^	0.62	Wolf *et al*. 2015^21^
sorghum	Sorghum	Cereals	0.44^87^	0.18^87^	0.86^93^	0.46	Wolf *et al*. 2015^21^
sorghumfor	Sorghum for forage and silage	Forage	0.95^86^	0.18^87^	0.35^21^	0.44	Wolf *et al*. 2015^21^
soybean	Soybeans	Oil Crops	0.42^87^	0.19^92^	0.88^93^	0.52	Wolf *et al*. 2015^21^
sugarbeet	Sugar beets	Sugar Crops	0.40^87^	0.43^87^	0.15^87^	0.41	Wolf *et al*. 2015^21^
sugarcane	Sugar cane	Sugar Crops	0.75^90,130,131^	0.18^87^	0.26^132^	0.41	Wolf *et al*. 2015^21^
sugarnes	Sugar crops, other	Sugar Crops	0.28^99^	0.18^112^	0.56^99^	0.41	Monfreda *et al*. 2008^20^
sunflower	Sunflower seed	Oil Crops	0.27^87^	0.06^87^	0.91^93^	0.62	Wolf *et al*. 2015^21^
swedefor	Swedes for fodder	Forage	0.95^86^	0.15^5^	0.13^5^	0.41	Wolf *et al*. 2015^21^
sweetpotato	Sweet potatoes	Roots & Tubers	0.53^101^	0.15^97^	0.20^97^	0.41	Wolf *et al*. 2015^21^
taro	Taro	Roots & Tubers	0.53^101^	0.15^97^	0.20^97^	0.41	Wolf *et al*. 2015^21^
triticale	Triticale	Cereals	0.50^87^	0.14^125,126^	0.90^87^	0.46	Wolf *et al*. 2015^21^
turnipfor	Turnips for fodder	Forage	0.95^86^	0.15^97^	0.13^5^	0.41	Wolf *et al*. 2015^21^
vegfor	Vegetables and roots for fodder	Forage	0.95^86^	0.15^5^	0.13^5^	0.41	Wolf *et al*. 2015^21^
vetch	Vetches	Pulses	0.95^86^	1.10^104^	0.35^21^	0.44	Wolf *et al*. 2015^21^
wheat	Wheat	Cereals	0.39^87^	0.20^87^	0.87^93^	0.46	Wolf *et al*. 2015^21^
yam	Yams	Roots & Tubers	0.53^101^	0.15^97^	0.20^97^	0.41	Wolf *et al*. 2015^21^
yautia	Yautia	Roots & Tubers	0.53^101^	0.15^97^	0.20^97^	0.41	Wolf *et al*. 2015^21^

Parameters were primarily compiled from two secondary sources: Monfreda *et al*.^20^ and Wolf *et al*.^21^.
